# Salicylate-Elicited Activation of AMP-Activated Protein Kinase Directly Triggers Degradation of C-Myc in Colorectal Cancer Cells

**DOI:** 10.3390/cells14040294

**Published:** 2025-02-17

**Authors:** Ana Laura S. A. Matos, Ashley J. Ovens, Emil Jakobsen, Diego Iglesias-Gato, Jacob M. Bech, Stine Friis, Lasse Kristoffer Bak, Gunvor I. Madsen, Jonathan S. Oakhill, Pietri Puustinen, José M. A. Moreira

**Affiliations:** 1 Department of Drug Design and Pharmacology, Faculty of Health and Medical Sciences, University of Copenhagen, 2100 Copenhagen, Denmark; 2 CAPES Foundation, Ministry of Education of Brazil, Brasília DF 70040-020, Brazil; 3 Metabolic Signalling Laboratory, St. Vincent’s Institute of Medical Research, Fitzroy, VIC 3065, Australiajoakhill@svi.edu.au (J.S.O.); 4 Department of Medicine, University of Melbourne, Parkville, VIC 3010, Australia; 5 Sino-Danish Center for Education and Research, Aarhus University, 8000 Aarhus, Denmark; 6 Department of Clinical Biochemistry, Copenhagen University Hospital-Rigshospitalet, 2600 Glostrup, Denmark; 7 Translational Research Center (TRACE), Copenhagen University Hospital-Rigshospitalet, 2600 Glostrup, Denmark; 8 Department of Pathology, Odense University Hospital, 5000 Odense, Denmark; 9 Cell Death and Metabolism, Danish Cancer Society Research Center (DCRC), 2100 Copenhagen, Denmark

**Keywords:** colorectal cancer, chemoprevention, aspirin, c-Myc degradation, molecular mechanisms

## Abstract

Aspirin has consistently shown preventive effects in some solid cancers, notably colorectal cancer. However, the precise molecular mechanisms underlying this positive effect have remained elusive. In this study, we used an azoxymethane-induced mouse model of colon carcinogenesis to identify aspirin-associated molecular alterations that could account for its cancer-preventive effect. Transcriptomic analysis of aspirin-treated mice showed a strong reduction in c-Myc protein levels and effects on the Myc-dependent transcriptional program in colonic cells. Proto-oncogene c-Myc cooperates with AMP-activated protein kinase (AMPK) to control cellular energetics. Here, we show that salicylate, the active metabolite of aspirin, reduces c-Myc protein expression levels through multiple mechanisms that are both AMPK dependent and independent. This effect is cell-type dependent and occurs at both the transcriptional and post-translational levels. Salicylate-induced AMPK activation leads to the phosphorylation of c-Myc at Thr400, as well as its destabilization and degradation. Our results reveal a complex, multilayered, negative effect of salicylate on c-Myc protein abundance and suggest that chronic depletion of c-Myc can counteract the neoplastic transformation of colorectal epithelium, underpinning the preventive effect of aspirin on colorectal cancer.

## 1. Introduction

Colorectal cancer (CRC) is the third most-common cancer worldwide, with an estimated 1.8 million new cases diagnosed in 2018, for both sexes combined [1]. CRC is a life-threatening disease with high incidence, morbidity, and mortality. Due to demographics and life-style changes, exposure to factors that are associated with a higher CRC risk, such as excessive alcohol intake, Western dietary patterns, obesity, and smoking [2,3,4,5,6,7], is ever increasing, and as a result, the incidence of CRC is expected to grow dramatically. The cancer burden of CRC is projected to increase from 1.9 million cases in 2020 to approximately 3.1 million cases in 2040, an increase of over 70% [1,8]. Current strategies for reducing CRC incidence and mortality are mainly based on secondary prevention initiatives, such as screening programs, with focus on early detection and removal of precancerous lesions [9]. But primary prevention strategies based on the use of pharmacological agents offer a promising alternative approach to control cancer [10,11].

Over the past three decades, a growing body of literature has emerged, based on multiple lines of evidence, ranging from cell-based data to observational epidemiological studies, suggesting aspirin and other nonsteroidal anti-inflammatory drugs (NSAIDs) can suppress carcinogenesis and delay development of bowel cancer [12,13]. Aspirin (acetylsalicylic acid) is a commonly prescribed NSAID, with anti-thrombotic and anti-inflammatory actions, and has attracted considerable attention as a potential chemopreventive agent [14]. Salicylate, the primary metabolite of aspirin, irreversibly acetylates and inactivates cyclooxygenase (COX) enzymes, inhibiting prostaglandin synthesis [15,16]. But salicylate modulates multiple additional molecular targets, many of which may also contribute to its anticancer effect on CRC. For example, salicylate affects nuclear factor kappa B (NF-κB) signaling [17,18,19], and it allosterically promotes the activation of 5′-AMP-activated protein kinase (AMPK) [20,21]. AMPK is a heterotrimeric serine/threonine protein kinase, consisting of a catalytic α subunit and two regulatory subunits (β and γ), which is activated in response to a decrease in the ATP:AMP ratio in order to preserve body energy homeostasis [22,23]. Accumulating evidence suggests a key role for AMPK in cancer initiation, by exerting tumor-suppressor functions, influencing inflammation, cell survival, and modulating cell-cycle arrest, and blocking the metabolic changes that take place during tumorigenesis [23,24,25,26,27]. Although multiple COX-dependent as well as -independent mechanisms have been proposed, the precise molecular events underpinning the anticancer effects of aspirin are yet to be unequivocally established [28]. Given the risks associated with long-term aspirin use, implementation of any aspirin chemoprevention strategy is likely to require patient stratification and targeting of individuals for whom the benefits outweigh the harms, which in turn necessitates a better understanding of aspirin’s mechanism of action. This study aimed to contribute mechanistic information that can further our understanding of the effectiveness of long-term aspirin intake for CRC prevention. We investigated molecular events linking salicylate-mediated AMPK activation and cancer prevention and show here an effect of salicylate on the stability of the c-Myc proto-oncogene mediated by AMPK activation.

## 2. Materials and Methods

### 2.1. Cell Culturing

DLD-1 (RRID:CVCL_0248), HCT116 (RRID:CVCL_0291), MCF7 (RRID:CVCL_0031), U2OS (RRID:CVCL_0042), COS-1 (RRID:CVCL_0223), and HEK293 (RRID:CVCL_0045) cells were obtained from the American Type Culture Collection (ATCC, Manassas, VA USA). Cells were grown in the recommended medium supplemented with 6%, or 10%, as appropriate, fetal bovine serum (FBS). Drugs were added at different concentrations and incubated from 24 up to 72 h according to the experiment to be performed. Mycoplasma testing was performed on a regular basis (Mycoplasma assay; Eurofins BioPharma, Glostrup, Denmark). Cell identity was verified by STR profiling (IdentiCell, Aarhus, Denmark).

### 2.2. Animal Studies

All animal experiments were conducted according to the Danish Guidelines for animal welfare and were approved by the Animal Experiments Inspectorate (permission nr. 2016-15-0201-00834). For tumor induction in the azoxymethane (AOM) murine model, 6-to-8-week-old female JCr mice were treated four times with 10 mg/kg of AOM once a week by intraperitoneal injection and sacrificed 6 months after the first AOM administration. Mice in the aspirin treatment group were administered aspirin for 6 months by adding acetylsalicylic acid to their diet (600 mg acetylsalicylic acid/kg Altromin 1324 regular diet; Brogaarden, Hørsholm, Denmark). A daily consumption of 2.5 g acetylsalicylic acid-enriched diet was used for dose calculation. This leads to an estimated daily intake of 60 mg/kg of aspirin. Tumors in the colon were excised under a dissecting microscope, and colonic tumor tissue was snap frozen. Assessment of tumor development was performed using endoscopy and scored as previously described [29]. Briefly, mice were examined by colonoscopy from expected onset at week 12 (3 months after the first AOM injection) using a Mainz Coloview system (Karl Storz, Tuttlingen, Germany), and from there on every month to follow the development of the tumors. The number of tumors was counted, and each tumor scored from 1 to 5 based on the burden relative to colon circumference [29].

### 2.3. Clinical Samples

A total of 20 patients presenting adenomatous polyps diagnosed and removed by colonoscopy in the period of 2002–2012 at Odense University Hospital were included in this study. Patients with intestinal polyposis syndromes or a prior history of colon cancer were excluded. All the adenomas were evaluated by a trained pathologist and classified as high-grade dysplasia. This study’s protocol was in accordance with the Declaration of Helsinki and was approved by the Regional Committee D on Health Research Ethics of the Capital Region of Denmark (j.nr. H-22022432) and granted exemption from obtaining informed consent (as per Section 10, Section 1, of the Committee Act).

### 2.4. Reagents and Antibodies

Cell culture reagents, such as media and FBS, were purchased from Invitrogen (Carlsbad, CA, USA). FuGENE 6 transfection reagent was purchased from Promega (Madison, WI, USA). Sodium salicylate, phenformin, 4-hydroxytamoxifen, antimycin A, carbonyl cyanide 4-(trifluoromethoxy) phenylhydrazone (FCCP), cycloheximide, MG132, oligomycin A, and rotenone were purchased from Sigma-Aldrich (St. Louis, MO, USA). Rabbit Acetyl-coA Carboxylase Antibody (cat #3662), Phospho-Acetyl-CoA Carboxylase (Ser79) Antibody (cat #3661), Phospho-AMPKα (Thr172) (D79.5E) Rabbit mAb (cat #2535), AMPKα1 Antibody (cat #5831), c-Myc Antibody (cat #9402), and Phospho-c-Myc (Ser62) (E1J4K) Rabbit mAb (cat #13748), phospho-PP2A alpha (Tyr307), and PP2A antibodies were from Cell Signaling Technologies (Danvers, MA, USA). AMPKα 1/2 total antibody (clone 34.2) (cat #ab80039) was from Abcam (Cambridge, UK). The mouse monoclonal antibody against CIP2A (2G10-3B5) was obtained from Santa Cruz Biotechnology (Dallas, TX, USA). Mouse monoclonal anti-HA antibody and ubiquitin antibody were acquired from Sigma-Aldrich (St. Louis, MO, USA) and Thermo Fisher Scientific (P4D1) (Waltham, MA, USA), respectively. Protein G sepharose (Fast Flow) and protein A sepharose (Fast Flow) were from GE Healthcare (Chicago, IL, USA). Recombinant full-length human protein AMPK (α1/β1/γ1), active (P47-10H-05), and GSK3β, active (G09-10G) were from SignalChem Biotech (Richmond, BC, Canada).

### 2.5. Western Blotting

Cells were plated at a density of 1 × 10^5^ cells/well in six-well culture plates and allowed to attach overnight. The next day, culture media were removed, and fresh warm media containing relevant compounds were added. Cells were exposed to relevant drugs at various concentrations, as indicated, and washed thrice with 1× phosphate buffered saline (PBS). Cells were then lysed in-plate and scraped using lysis buffer (10 mM Tris-HCL pH 7.4, 150 mM NaCl, 15% glycerol, and 1% Triton X-100) with protease inhibitors (cOmplete™ Protease Inhibitor Cocktail, Merck, St. Louis, MO, USA). The protein samples were diluted with MilliQ water and 4× Laemmli sample buffer (1/4 of volume, Sigma-Aldrich, St. Louis, MO, USA) containing 0.1% DTT (Sigma-Aldrich). Samples containing 15 µg of total protein were incubated at 95 °C for 10 min and placed on ice until loaded. Samples were loaded in 15-well gels (4–15% Mini-PROTEAN TGCTM gel; Bio-Rad, Hercules, CA, USA), resolved, and immunoblotted with relevant antibodies. To estimate protein size, a molecular-weight standard was loaded on each gel (Precision Plus Protein Dual; Bio-Rad, USA). Immunoblot images were quantified using ImageJ v.1.54i software (U. S. National Institutes of Health, Bethesda, MD, USA; https://imagej.net/ij/). Bands were normalized to loading controls GAPDH or β-actin, and for activated proteins, expression values were normalized again to total protein values to calculate activation ratios.

### 2.6. Quantitative Real-Time PCR

For qRT-PCR studies, cells were plated at a density of 1 × 10^5^ cells/well in six-well culture plates and allowed to attach overnight. The next day, wells were divided into treatment groups with three wells per group. After treatment, samples were washed twice in cold sterile PBS. Total RNA was extracted using TRIzol reagent according to the manufacturer’s instructions (Invitrogen, Carlsbad, CA, USA). The expression levels of *MYC* and *GAPDH* genes were determined by qRT-PCR on a LightCycler^®^ 480 Real-Time PCR System (Roche, Basel, Switzerland). QPCR primer pairs for human MYC (#HP206146) and human GAPDH (#HP205798) were from OriGene Technologies (Rockville, MD, USA). Amplification conditions followed manufacturer’s instructions (OriGene Technologies). For normalization of the amount of sample RNA added to each reaction, threshold cycle (Ct) values of GAPDH were subtracted from the corresponding Ct value of MYC. Results are presented as normalized ratios relative to control.

### 2.7. In Vitro C-Myc Phosphorylation by AMPK for LC-MS/MS Analysis

Recombinant human proto-oncogene c-Myc protein was purchased from RayBiotech (cat #230-00580; RayBiotech, Peachtree Corners, GA, USA). Recombinant c-Myc was phosphorylated in vitro by a bacterially expressed, purified, and CaMKK2-treated AMPK-hα1/myr-β1/hϒ1 complex (10:1, *v*:*v*). An in vitro kinase assay was performed at 30 °C for 30 min in 50 mM HEPES (pH 7.4), 2 mM MgCl_2_, 0.2 mM ATP, and 1 mM DTT. The reaction was stopped by adding SDS-PAGE sample buffer and boiling the samples for 5 min. The samples were run on SDS-PAGE for visualization and subsequent in-gel digestion of phosphorylated c-Myc. A 12% (*w*/*v*) polyacrylamide gel containing 50 μmol/L Phos-Tag acrylamide was used to visualize the differential migration of the phosphorylated c-Myc protein bands following the in vitro kinase assay.

### 2.8. In-Gel Trypsin Digestion of C-Myc and LC-MS/MS Analysis

Coomassie Brilliant Blue stained c-Myc bands were excised from the PAGE gel. Excised bands were further destained with 50 mM Triethyl Ammonium Bicarbonate (TEAB) (50%)/acetonitrile (50%) overnight and subsequently spent 30 min on a rotation device. Gel plugs were dehydrated for 30 min with 100% acetonitrile. Dehydrated gel plugs were reduced with 10 mM Tris (2-Carboxyethyl) phosphine (TCEP) for 45 min at 55 °C and alkylated with 55 mM iodoacetamide at room temperature in the dark for 30 min. Gel pieces were washed 3 times with 50 mM TEAB for 10 min each on a rotation device before they were dehydrated with 100% acetonitrile. Dehydrated gel plugs were digested with trypsin (Sigma-Aldrich; cat # T7575) dissolved in 25 mM TEAB at 37 °C overnight. Digested tryptic peptides were freeze-dried and resuspended in 0.1% (*v*/*v*) formic acid and analyzed by LC-MS/MS using a Q-Exactive plus mass spectrometer (Thermo Scientific, MA, USA) fitted with a nanoflow reverse-phase HPLC (Ultimate 3000 RSLC, Dionex, Sunnyvale, CA, USA). The nano-LC system was equipped with an Acclaim Pepmap nano-trap column (Dionex—C18, 100 Å, 75 μm × 2 cm) and an Acclaim Pepmap RSLC analytical column (Dionex—C18, 100 Å, 75 μm × 50 cm). Typically, for each LC-MS/MS experiment, 5 μL of the peptide mix was loaded onto the enrichment (trap) column at an isocratic flow of 5 μL/min of 3% (*v*/*v*) acetonitrile containing 0.1% (*v*/*v*) formic acid for 6 min before the enrichment column was switched in-line with the analytical column. The eluents used for the LC were 0.1% (*v*/*v*) formic acid (solvent A) and 100% acetonitrile/0.1% formic acid (*v*/*v*) (solvent B). The gradients used were 3% B to 25% B for 23 min, 25% B to 40% B in 2 min, and 40% B to 80% B in 2 min, and maintained at 85% B for the final 2 min before equilibration for 9 min at 3% B prior to the next analysis. All spectra were acquired in positive mode with full-scan MS spectra scanning from *m*/*z* 375–1400 at 70,000 resolution with an AGC target of 3 × 10^6^ with a maximum accumulation time of 50 ms. A lockmass value of 445.120024 was used, the 15 most-intense peptide ions with charge states ≥ 2–5 were isolated with an isolation window of 1.2 *m*/*z* and fragmented with a normalized collision energy of 30 at a resolution of 35,000 resolution with an AGC target of 1 × 10^5^ and a maximum accumulation time of 120 ms. Underfill threshold was set to 2% for triggering of precursor for MS2. Dynamic exclusion was activated for 30 s. Mass spectrometric raw data were searched using the Mascot search algorithm against the human SwissProt database. Cysteine carbamidomethylation was searched as a fixed modification, whereas oxidation of methionine and phosphorylation of serine, threonine, and tyrosine were searched as variable modifications.

### 2.9. Extracellular Flux Analysis

To assess the effect of sodium salicylate on mitochondrial respiration in HCT116 cells, the cells were subjected to the Seahorse Mitochondrial stress test according to the manufacturer’s protocol (Seahorse Bioscience, Agilent Technologies, Santa Clara, CA, USA). Twenty-four hours before an experiment, cells were seeded at 30,000 cells/well in a 96-well Seahorse plate. One hour before the experiment, the cells were washed and placed into Seahorse assay medium (DMEM with 3 mg/L phenol red and 5 mM glucose, pH 7.4) with sodium salicylate (1 mM or 3 mM) or vehicle control and kept at 37 °C. After baseline measurements, oligomycin A (final concentration of 1 µM), FCCP (final concentration of 1 µM), and antimycin A/rotenone (final concentration of 0.5 µM for both) were sequentially injected according to the protocol. Three measurements were performed between each injection. Oxygen consumption rate (OCR) measurements were analyzed as described by Zhang and colleagues [30]. First, the oxygen consumption rate (OCR) measured as the last point after the antimycin A/rotenone injection was subtracted from all other measurements to subtract non-mitochondrial respiration. Proton leaking was calculated as post-oligomycin OCR as percent of basal OCR, respiration related to ATP production was calculated by subtracting the post-oligomycin OCR from basal OCR and presented as percent of basal OCR, and the maximal respiratory capacity was calculated as post-FCCP OCR as percent of basal OCR. Each condition was performed with 4 technical replicates in each experiment. The data are presented as the mean of four different experiments. Statistical analyses were performed employing one-way ANOVA followed by Dunnet’s multiple comparisons test.

### 2.10. Lentiviral-Mediated AMPK and MYC Expression and Purification

Hemagglutinin (HA)-tagged recombinant c-Myc and HA empty vector plasmids were transiently transfected into HEK293 cells. The cells were lysed with RIPA buffer and the lysate was cleared by centrifugation. Anti-HA affinity gel was used to purify HA-tagged proteins. Immunopurified HA-tagged proteins were washed extensively in RIPA buffer and washed further with a kinase buffer, thrice (50 mM Tris pH 7.5, 100 mM MgCl, 0.2 mM EGTA, and 1 mM DTT). HA-tagged proteins were released from the beads by washing with HA peptide, and the supernatant was immediately used in a kinase assay reaction. For the in vitro kinase reaction, the HA-purified proteins were either immobilized to HA beads or released before the kinase reaction by using an excess amount of the HA peptide. For the kinase assay reaction, recombinant purified AMPK complex with HA-purified c-Myc substrate was mixed with kinase reaction buffer [50 mM Tris pH 7.5, 100 mM MgCl, 0.2 mM EGTA, and 1 mM DTT (ATP 0.2 mM, 0.2 μg BSA α-32 P-ATP, 3000 Ci/mmol)] at 30 °C for 15 min. The reaction was terminated by adding SDS sample buffer, and the samples were subjected to SDS–PAGE gel separation. Proteins were transferred onto a nitrocellulose filter (Bio-Rad). The filters were washed extensively with PBS, dried, and the incorporation of labeled phosphate on the peptides was quantified using a Typhoon 9410 imaging reader (Amersham Biosciences, Amersham, UK). Protein-incorporated α-32 P-ATP, 3000 Ci/mmol was quantitated and visualized with FujiFilm MultiGauge version 3.2.

### 2.11. Immunofluorescence Staining and Analysis

Cells were grown on glass coverslips, fixed in 4% paraformaldehyde for 20 min, permeabilized in 0.5% Triton X-100 in PBS for 10 min, and washed 3 times with PBS for 5 min each time. All steps were performed on ice. The cells were blocked with 2% BSA in PBS containing 0.1% Tween-20 for 1 h, at room temperature or overnight at 4 °C. Cells were then incubated at room temperature with primary antibodies for 1 h, washed four times in blocking buffer for 10 min, and then incubated at room temperature with a fluorophore-conjugated secondary antibody for 1 h. After incubating with the secondary antibody, the cells were washed three times in 0.1% Tween-20 in PBS, before mounting. Slides were imaged with a Nikon A1R confocal microscope. Images were collected and processed with Nikon software, NIS-elements C. Images were acquired at 20× magnification and collected sequentially for multicolor imaging. The levels of nuclear fluorescence (DAPI and c-Myc, blue and red channels, respectively) were determined from the fluorescence microscopy images using ImageJ v.154i.

### 2.12. Immunohistochemistry (IHC) and Quantitative Assessment of IHC Staining

Three µm sections cut from FFPE blocks of tissue samples were mounted onto glass slides. The sections were deparaffinized with xylene and rehydrated in a graded series of ethanol concentrations. Antigen retrieval was performed by heating sections for 10 min in a microwave with Envision Flex Target Retrieval Solution Low pH or High pH (Agilent Technologies), depending on the antibody. Sections were blocked with 1% peroxide for 10 min, permeabilized in TBS + Triton X-100, and incubated overnight at 4 °C with an anti-c-Myc antibody (clone EP121) or Phospho-AMPKα (Thr172) (40H9) rabbit monoclonal antibody (Cell Signaling Technology) and diluted in Antibody Diluent (Agilent). The next day, the sections were washed in TBS + Triton X-100, incubated with HiDef detection amplifier (Cell Marque, Sigma-Aldrich), washed in TBS + Triton X, incubated with HiDef Detection HRP Polymer Detector (Cell Marque, Sigma-Aldrich), and washed and visualized with diaminobenzidine (DAB) chromogen (Agilent). Sections were counterstained with hematoxylin. To allow accurate comparisons, the incubation and development times were standardized. Normal rabbit serum instead of primary antibody was generally used as a negative control. Slides were scanned using a NanoZoomer-XR Digital slide scanner (Hamamatsu Photonics, Shizuoka, Japan), and whole slide images were analyzed with QuPath v.0.5.0 digital pathology image analysis software (https://qupath.github.io), an open source imaging platform. Negative, weak, moderate, and strong immunostaining thresholds were set based upon mean DAB optical densities, and scores were calculated from the intensity of the staining.

### 2.13. Gene Expression Analysis

RNA was purified from frozen tissues using the RNeasy plus kit (Qiagen, Hilden, Germany). Gene expression profiles were determined using the Mouse Agilent 4 × 44 K v2 gene expression microarray (Agilent Technologies). The single-color microarray data were analyzed by Significance Analysis of Microarrays (SAM) using the MeV software suit (http://webmev.tm4.org/). Differential expression was filtered based on an FDR-corrected *p*-value of less than 0.1 (corresponding to an FDR of 10%). Enrichment of transcription factor binding sites on gene promoters from differentially expressed genes between histologically normal tissues from AOM- and AOM + salicylate-treated mice were analyzed using the Molecular Signatures Database (MsigDB) [31,32].

### 2.14. Statistical Analysis

All experiments were repeated at least in triplicate, independently of each other. Unless otherwise indicated, in all cases, one-way ANOVA was adopted to evaluate differences between groups with significance defined as *p* < 0.05.

## 3. Results

### 3.1. Acetylsalicylic Acid Affects the Myc-Dependent Transcriptional Program

**Figure 1:**
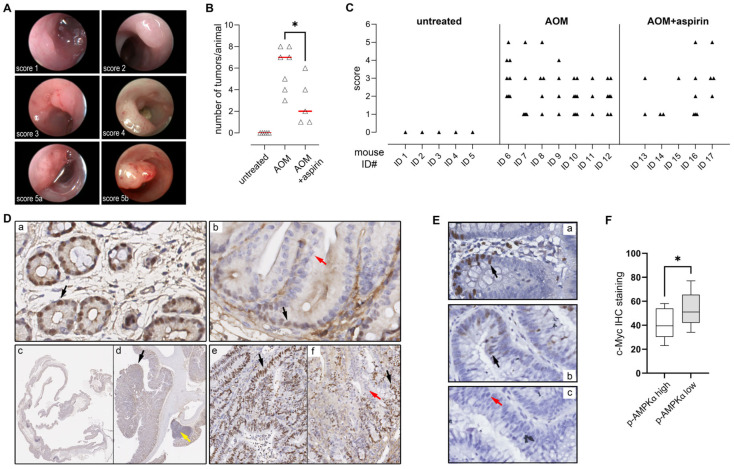
Aspirin has a preventive effect on mice with chemically induced CRC lesions. (**A**) Representative images of lesions in AOM-treated mice. Lesions were examined by colonoscopy. Tumors were counted, and each tumor scored from 1 to 5 based on the burden relative to colon circumference, according to [29]. (**B**) Tumor number and (**C**) tumor score in untreated, AOM-treated, and AOM+aspirin-treated mice. Significance was evaluated with unpaired Student’s *t*-test. (**D**) immunohistochemical analysis of c-Myc in normal colonic tissue in AOM-treated (subpanel (**a**)) and AOM+aspirin-treated mice (subpanel (**b**)). The entire intestinal tract was collected using the Swiss-rolling technique (subpanel (**c**)), and lesions (subpanel (**d**)) were identified and analyzed, both in AOM-treated (subpanel (**e**)) and AOM+aspirin-treated mice (subpanel (**f**)). Colonic cells with expression of c-Myc are indicated by black arrows and cells with no expression by red arrows. Yellow arrow indicates immune cells. Original magnification was 40× for subpanels (**a**,**b**), 4× for subpanels (**c**,**d**), and 20× for subpanels (**e**,**f**). (**E**) Histological analysis of colorectal adenomas. Depicted are representative c-Myc stainings of HG adenomas (subpanels (**a**–**c**): c-Myc high, intermediate, and low expression, respectively). Original magnification was 40× for all subpanels. (**F**) Bar graph represents quantification of c-Myc-positive cells (n = 20 samples; * *p* < 0.05, Student’s *t*-test) in samples with high or low *p*-AMPKα T172 immunoreactivity.

Aspirin (acetylsalicylic acid) is a promising drug for the chemoprevention of colorectal cancer (CRC), with observational studies and large-scale randomized controlled intervention trials showing it to have a protective effect. Although multiple COX-dependent and -independent mechanisms have been proposed, the precise mechanism underpinning aspirin’s cancer-preventive effect is still unclear [14]. We used the azoxymethane (AOM)-induced mouse model of colon carcinogenesis, which mimics sporadic colon tumor development, to identify acetylsalicylic acid-associated molecular alterations that could account for its CRC chemopreventive effect. As expected, regular administration of acetylsalicylic acid significantly reduced both colorectal tumor incidence and burden in AOM-treated mice (Figure 1A–C).

The average number of tumors present in each animal was significantly higher in AOM-only-treated mice than in AOM + acetylsalicylic acid-treated mice (Figure 1B). Also, the average tumor score was reduced in acetylsalicylic acid-treated mice (Figure 1C). To identify acetylsalicylic acid-induced changes, we compared the gene expression profiles of histologically normal colonic tissue samples collected from AOM-treated and AOM+salicylate-treated mice. Significance Analysis of Microarrays (SAM) identified 632 gene probes that changed significantly (FDR < 0.05, Supplementary Table S1). GO analysis of these genes did not result in a meaningful grouping of biological processes or pathways. However, all the identified deregulated genes were expressed at lower levels in the acetylsalicylic acid-treated animals, suggesting the involvement of a transcription factor. We investigated whether the 632 gene probes that changed significantly were enriched for specific transcription factor binding sites using the Molecular Signature Database from the Broad Institute (searching database: TFT_Legacy). We found that there were 32 genes with canonical Myc binding sites (*p*-val: 3.78 × 10^−6^ and FDR q-val: 1.09 × 10^−4^), suggesting acetylsalicylic acid treatment affected the Myc-dependent transcriptional program in colonic cells.

Proto-oncogene c-Myc is an essential determinant of cell proliferation and a critical mediator of the early stages of neoplasia in CRC, following loss of APC [33]. Salicylate was previously shown to decrease c-Myc protein levels in colon cancer cells [34,35]. To determine whether acetylsalicylic acid decreased c-Myc levels in vivo, we examined c-Myc expression in colonic tissue samples collected from our AOM-treated and AOM+ acetylsalicylic acid-treated mice (Figure 1D). Immunohistochemical analysis of c-Myc showed strong nuclear expression in the proliferating cells of the crypts in AOM-treated mice (Figure 1D, subpanel a, black arrow), as expected [36]. In AOM+ acetylsalicylic acid-treated mice, expression of c-Myc was still restricted to crypt cells (Figure 1D, sub-panel b, black arrow), but staining was weak, or undetectable, and not strictly nuclear, consistent with strongly decreased levels of c-Myc. This effect was apparent both in histologically normal tissue and in lesions (Figure 1D; subpanels c through f).

### 3.2. Salicylate Promotes Activation of AMPK and Degradation of C-Myc

**Figure 2:**
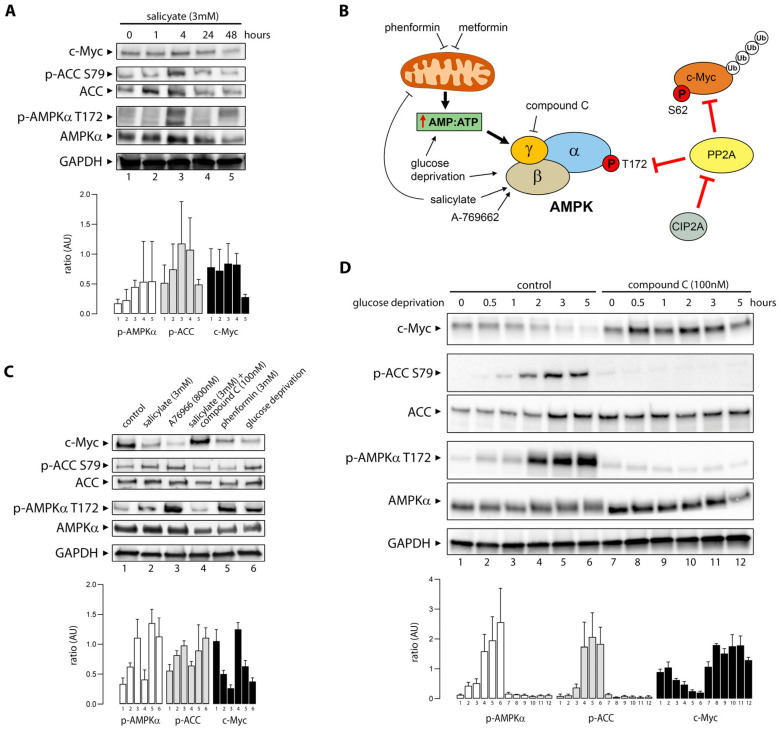
AMPK activation causes loss of c-Myc. (**A**) Exposure of HCT116 colon cancer cells to 3 mM salicylate for 1, 4, 24, or 48 h (lanes 2 through 5) elicited AMPK activation, evaluated by phosphorylation of AMPKα at T172, followed by loss of c-Myc expression. Acetyl-CoA carboxylase (ACC) phosphorylation (S79) was used as a marker of functional AMPK activation. (**B**) Schematic illustration of the molecular mechanisms leading to the activation or inhibition of AMPK. Modulators of AMPK used in this study, negative as well as positive, and their expected mechanism of action are represented. (**C**) Decreased levels of c-Myc are associated with AMPK activation. HCT116 cells were grown in complete medium for 48 h in the absence (lane 1) or presence of salicylate (lane 2), the synthetic AMPK activator A76966 (lane 3), phenformin (lane 5), or grown in glucose-free medium for 8 h (lane 6). Cells were also treated with a combination of salicylate (3 mM) and compound C (dorsomorphin 100 nM) for 48 h (lane 4). (**D**) HCT116 cells were cultured in glucose-free medium for up to 5 h in the absence (lanes 1 through 6) or presence of compound C (lanes 7 through 12). Representative western blots and quantification graphs of triplicate experiments are shown. Data are presented as mean ± SEM, n = 3.

Salicylate, the primary metabolite of aspirin, modulates multiple molecular targets. Among others, it elicits activation of AMPK [20,21], and it has been shown to cause downregulation of c-Myc [34]. Although Myc and AMPK play antagonistic regulatory roles in cellular metabolism, their relationship is quite complex with respect to carcinogenesis as they are highly interconnected and appear to engage in significant crosstalk [37]. We investigated the effect of salicylate exposure on AMPK and c-Myc (Figure 2A) in HCT116 colon cancer cells left untreated or exposed to 3 mM salicylate for 1 h, 4 h, 24 h, or 48 h.

Phosphorylation of the bona fide AMPK substrate acetyl-CoA carboxylase (ACC) was used as a measure of functional AMPK activation. Exposure of HCT116 cells to salicylate resulted in AMPK activation, evaluated by increased phosphorylation of AMPKα at Thr172 (p-AMPKα T172), and of the AMPK kinase substrate ACC at Ser79 (p-ACC S79) (Figure 2A). After 48 h of exposure to salicylate, levels of c-Myc protein decreased by more than three-fold (Figure 2A, lane 5).

AMPK is a regulator of cellular energy homeostasis that can be activated, directly or indirectly, by a variety of physiological stimuli and pharmacological inducers, leading to the modulation of multiple downstream effectors (Figure 2B). Salicylate is a direct activator of AMPK binding the allosteric drug and metabolite-binding site (ADaM) [20], but it is also thought to uncouple mitochondrial respiration [38], which in turn can lead to increased AMP:ATP ratios and AMPK activation. To establish whether decreased levels of c-Myc were associated with functional AMPK activation, we examined the effect of multiple stimuli that activate AMPK, such as the synthetic direct activator A76966, which binds the ADaM site [39], and phenformin, a mitochondrial Complex I inhibitor. We found that both A76966 and phenformin decreased c-Myc protein levels (Figure 2C, lanes 3 and 5, respectively). This effect was also observed with glucose deprivation, a physiological stimulus (Figure 2C, lane 6). Conversely, blocking activation of AMPK with compound C (dorsomorphin; 100 nM), a potent AMPK inhibitor [40], prevented downregulation of c-Myc expression by salicylate (Figure 2C, lane 4).

The activation of AMPK by glucose withdrawal can occur via the canonical energy-sensing mechanism and through an AMP/ADP-independent mechanism [41]. We deprived HCT116 colon cancer cells of glucose for up to five hours (Figure 2D), resulting in a time-dependent decrease in c-Myc expression. To investigate the specific requirement for AMPK in c-Myc downregulation during glucose starvation, compound C (dorsomorphin; 100 nM) was used concomitantly to block AMPK activation. AMPK was activated 30 min after glucose withdrawal, determined by the increase in levels of p-AMPKα T172 and p-ACC S79. C-Myc was destabilized with delayed but similar kinetics to AMPK activation, measured by AMPK and ACC phosphorylation, and after five hours following glucose withdrawal, c-Myc protein levels were below the detection limits of the assay. Addition of compound C prior to glucose starvation strongly inhibited phosphorylation of p-AMPK T172 and the AMPK substrate p-ACC S79, indicating that AMPK kinase activity was inhibited. Addition of compound C prevented c-Myc depletion, indicating that AMPK plays a role in c-Myc expression during glucose starvation (Figure 2D).

**Figure 3:**
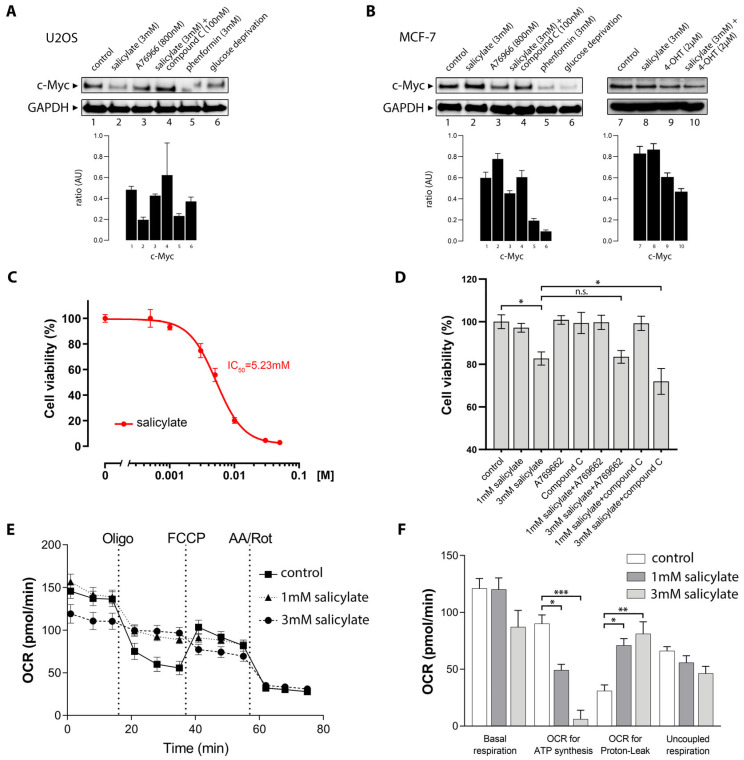
Salicylate affects multiple cell types and has diverse effects. (**A**) U2OS osteosarcoma cells and (**B**) MCF-7 breast cancer cells were grown in complete media for 48 h in the presence of salicylate (lane 2, (**A**,**B**), respectively), the synthetic activator A76966 (lane 3, (**A**,**B**), respectively), phenformin (lane 5, (**A**,**B**), respectively), or in glucose-free media for 8 h (lane 6, (**A**,**B**), respectively). Cells were also treated with a combination of salicylate (3 mM) and compound C (dorsomorphin 100 nM) for 48 h (lane 4, (**A**,**B**), respectively). In MCF-7 cells, the expression of c-Myc was significantly decreased by 4-OHT alone (lane 9) and even more by a combination of salicylate and 4-OHT (lane 10), compared with the negative control (lane 7). Glyceraldehyde-3-phosphate dehydrogenase (GAPDH) was used in all cases as loading control for protein normalization. (**C**) Cell viability of HCT116 cells was determined by MTT assay after treatment with indicated concentrations of salicylate for 48 h. IC_50_ for salicylate was determined using Prism v.9 (GraphPad Software, San Diego, CA, USA) based on the changes in HCT116 cell viability. (**D**) Cell viability was determined after 48 h in the presence of 1 mM or 3 mM of salicylate, the synthetic activator A76966, compound C (dorsomorphin: 100 nM), or combinations of these drugs. Mean values ± SEMs are shown. * *p* < 0.05; ** *p* < 0.01; *** *p* < 0.001; n.s.: not significant. (**E**,**F**) Seahorse extracellular flux analysis of the oxygen consumption rate (OCR) shows respiratory changes in HCT-116 cells brought about by sodium salicylate treatment. (**E**) Seahorse injection series analysis with OCR data presented for one experiment. (**F**) Calculated parameters from 3 independent experiments include ATP-linked respiration, proton leak, basal respiration, and maximal respiratory capacity. Basal respiration significantly increased when cells were treated with sodium salicylate (1 or 3 mM). Treatment with sodium salicylate caused an increased proton leak (uncoupling) and a decreased percental amount of oxygen being used for ATP production. The maximal respiratory capacity was lowered when treated with sodium salicylate, which suggests a compromised mitochondrial function.

To determine whether the effect of salicylate on AMPK activation and c-Myc degradation was a general effect, we investigated the association between AMPK activation and c-Myc levels in U2OS osteosarcoma cells and MCF-7 breast cancer cells (Figure 3A and Figure 3B, respectively).

Activation of AMPK, be it through exposure to salicylate, to the synthetic AMPK activator A76966 [39], phenformin (a biguanide), or through glucose deprivation, in all cases resulted in the reduction of c-Myc protein expression levels in U2OS osteosarcoma cells (Figure 3A). In MCF-7 breast cancer cells, however, salicylate did not decrease c-Myc protein levels, even though AMPK activation by A76966, phenformin, or glucose deprivation caused a reduction in the levels of c-Myc (Figure 3B). These data suggest that some form of compensatory mechanism may take place in MCF-7 cells, given that salicylate activated AMPK. Since *MYC* is a well-known estrogen-regulated gene [42], it was conceivable that downregulation of c-Myc by salicylate was offset by estrogen receptor-mediated upregulation of *MYC* gene expression. When treated with 4-hydroxy-tamoxifen (4-OHT), a selective estrogen receptor modulator, c-Myc expression was significantly decreased in MCF-7 cells (lane 9, Figure 3B). When 4-OHT was combined with salicylate, c-Myc expression was further decreased (lane 10, Figure 3B), consistent with a compensatory effect of ER signaling in MCF-7 cells.

### 3.3. Salicylate Reduces the Cell Viability of CRC Cells Through an AMPK-Independent Mechanism

Aspirin has been reported to inhibit cell growth and have a cytotoxic effect through multiple mechanisms, including c-Myc downregulation, proteasomal dysfunction, and induction of autophagy, among others [34,43,44]. We assessed the effect of salicylate on HCT116 cell viability and found that exposure of HCT116 cells to salicylate for 48 h caused a dose-dependent decrease in cell viability, with an IC_50_ of 5.23 mM (Figure 3C). The decrease in HCT116 cell viability caused by salicylate exposure was AMPK-independent, given that addition of A76966 to salicylate showed no further significant effect, positive or negative, on HCT116 cell viability (Figure 3D). Conversely, addition of compound C to salicylate, which would be expected to reduce the effect of salicylate on cell viability, showed an additive effect on decreased HCT116 cell viability (Figure 3D). This paradoxical effect may arise from the combinatorial treatment inducing mitochondrial dysfunction and synergistically enhancing both autophagic and apoptotic cell death through contextual AMPK-independent mechanisms, consistent with the previous findings [45,46].

### 3.4. Salicylate Affects the Respiratory Capacity of CRC Cells

Salicylate binds directly to AMPK at a binding pocket termed the ADaM site, which is also the target of synthetic drugs, such as A-769662, triggering allosteric activation of AMPK [21]. But salicylate has also been reported to affect cellular energy status and uncouple and inhibit mitochondrial electron transport [47], which in itself can activate AMPK [48]. We investigated whether salicylate affected cellular respiration in HCT116 cells. Treatment of HCT116 cells with salicylate (3 mM) significantly decreased basal respiration to 87.2 oxygen consumption rate (OCR), compared to 121.0 OCR in untreated cells (*p* = 0.042; Figure 3E). Proton leak accounted for 30.9% of the baseline OCR in untreated cells; however, in cells treated with sodium salicylate (1 mM or 3 mM), the proton leak significantly increased to 71.1% (*p* = 0.038) or 81.1% (*p* = 0.014) of the baseline, respectively (Figure 3F). In untreated cells, ATP production accounted for 90.2% of the baseline OCR, whereas in cells treated with sodium salicylate (1 mM or 3 mM), the oxygen consumption accounting for ATP production decreased to 49.2% (*p* = 0.043) and 6% (*p* = 0.020), respectively (Figure 3F). Salicylate (3 mM) also significantly decreased the maximum respiratory capacity in HCT116 cells from 66.1% to 46.2% (*p* = 0.017) of baseline (Figure 3F).

### 3.5. Activated AMPK Phosphorylates Ser-67, Ser-373, and Thr-400 Residues in C-Myc

C-Myc protein stability is tightly regulated at the cellular level in a phosphorylation-dependent manner. Multiple kinases have been implicated in c-Myc phosphorylation, including mitogen-activated protein kinase (MAPK), c-Jun N-terminal kinase (JNK), cyclin-dependent kinase 1 (CDK1), and casein kinase II (CK2) [49]. AMPK is a serine/threonine kinase able to regulate a large network of downstream effectors by directly phosphorylating them [50]. It was plausible that activated AMPK could bind and phosphorylate c-Myc directly, destabilizing it.

**Figure 4:**
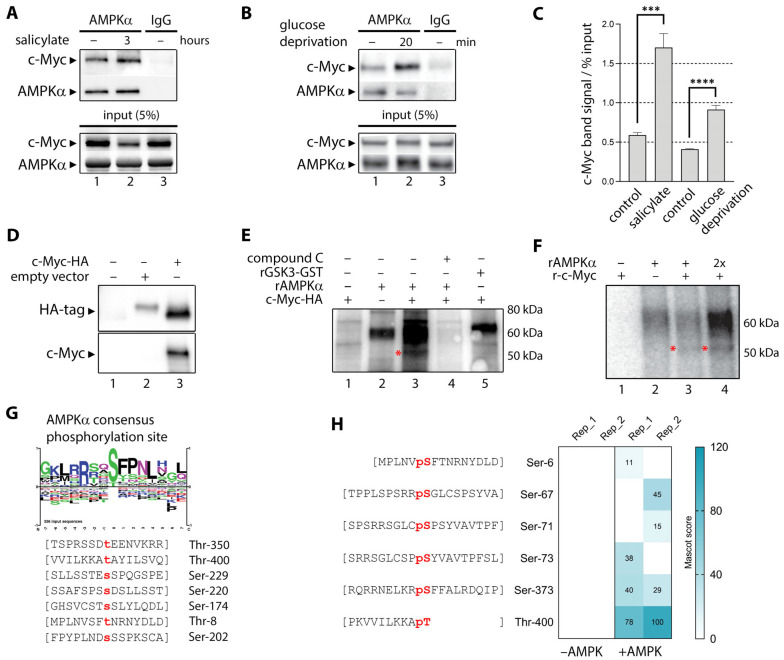
Dynamic interaction between AMPK and c-Myc regulates phosphorylation of c-Myc. Endogenous protein complexes were immunoprecipitated from HEK293 cell lysates with an AMPK1/2 antibody before and after (**A**) exposure to salicylate (3 mM for 3 h) or (**B**) glucose deprivation (20 min). Immunoprecipitated proteins were analyzed by immunoblotting, as indicated. Normal serum IgG served as a negative control. Immunoblots of the corresponding cell lysates are shown in the lower panel (input 5%). (**C**) Quantitation of three replicate experiments. Conditions were compared using Student’s *t*-test and the results are presented. *** *p* < 0.001; **** *p* < 0.0001. (**D**) Immunoblotting of purified c-Myc-HA-tagged protein used for the in vitro kinase reaction. Cells were mock-transfected (lane 1) or transfected with a control HA-tag vector (lane 2), or recombinant c-Myc-HA (lane 3), and proteins immunopurified using the HA tag. Immunoblotting with HA-antibodies (upper panel) and c-Myc antibodies (lower panel) confirmed the identity of purified proteins. (**E**,**F**) AMPK phosphorylates c-Myc in vitro. In vitro kinase reaction was performed with (**E**) HEK293-expressed c-Myc-HA purified protein (lane 1), recombinant full-length active human AMPK complex (α1/β1/γ1) (lane 2), or a combination of both (lane 3). Addition of compound C to AMPK complex prior to the kinase reaction abolished phosphorylation of substrates in reaction. Recombinant GSK3β (lane 5) was used as control of reaction specificity. (**E**) Bacterially expressed rc-Myc protein (lane 1), active human AMPK complex (α1/β1/γ1) (lane 2), or a combination of both (lanes 3 and 4, at 1x and 2x amounts of AMPK). The * symbol in the autoradiograph indicates the presence of a phosphorylated substrate compatible with c-Myc. (**G**) AMPK substrate consensus sequence (upper panel). Lower panel shows the AMPK motifs present on the c-Myc protein ranked by score. (**H**) Phosphorylated peptides identified by in-gel tryptic digestion and mass spectrometry of in vitro phosphorylation of recombinant c-Myc with AMPK-α1/β1/ϒ1 complex (+AMPK). A mock reaction lacking AMPK complex (−AMPK) was used as control. Phosphorylated residues are shown in red. Results from two independent experiments are shown.

We immunoprecipitated (IP) the AMPK protein complex with an AMPKα antibody from human HEK293 embryonic kidney cells before and after salicylate exposure (3 h). We found that c-Myc co-immunopurified with the AMPK complex (Figure 4A, lane 1), and that the amount of AMPK-associated c-Myc significantly increased after treatment with salicylate (Figure 4A, lane 2). Quantification of band signals and normalization to input lysate showed a very significant increase in co-IP c-Myc (*p* < 0.001; Figure 4B). AMPK was then activated by glucose deprivation for 20 min, and AMPK-associated c-Myc was investigated by co-IP (Figure 4C). As with salicylate, AMPK activation increased levels of AMPK co-purified c-Myc. Quantitation of co-purified c-Myc in immunopurified AMPK complexes showed a significant increase in association (*p* < 0.0001; Figure 4B). We then purified the recombinant HA-tagged c-Myc protein by transient expression in HEK293 cells. Protein purity was evaluated by Coomassie staining and immunoblotting with c-Myc and HA-tag antibodies (Figure 4D). Recombinant AMPK (rAMPKα) was then used to phosphorylate rc-Myc-HA in vitro using a mix of cold and γP^32^ labeled ATP as substrate (Figure 4E). HA-purified c-Myc alone did not show any substantial co-purifying kinase activity (Figure 4E, lane 1). When rAMPKα was added to the reaction, we observed a band consistent with autophosphorylated AMPKα (65 kDa), and a discrete band the size of c-Myc (asterisk; Figure 4E). This band was not present in the reactions with only rc-Myc-HA or rAMPKα (Figure 4E, lanes 1 and 2, respectively). Addition of compound C to the reaction abolished the presence of the bands consistent with c-Myc (55 kDa) and autophosphorylated AMPKα (65 kDa). Recombinant GSK3-GST (rGSK3-GST; Figure 3D, righthand panel) was used as a positive control for the in vitro phosphorylation reaction (three independent repeats).

To identify substrate motifs in c-Myc that may be recognized by AMPK, we matched the optimal AMPK substrate consensus motif (Baso_ST_kin group motif, AMP kinase motif) against the c-Myc protein sequence (UniProt accession P01106) [51]. We found seven putative sites that matched the AMPK consensus motif: Thr8, Ser174, Ser202, Ser220, Ser229, Thr350, and Thr400 (Figure 4G). To establish whether any of these sites can be phosphorylated by AMPK, we performed an in vitro kinase assay with recombinant c-Myc and purified AMPK complex (hα1/myr-β1/hγ1) followed by in-gel tryptic digestion and mass spectrometry. We identified six phosphopeptides, of which Thr400 consequently showed high levels of phosphorylation (Figure 4H).

### 3.6. Salicylate Regulates the Expression of C-Myc Protein in Part Through the Proteosome Pathway

**Figure 5:**
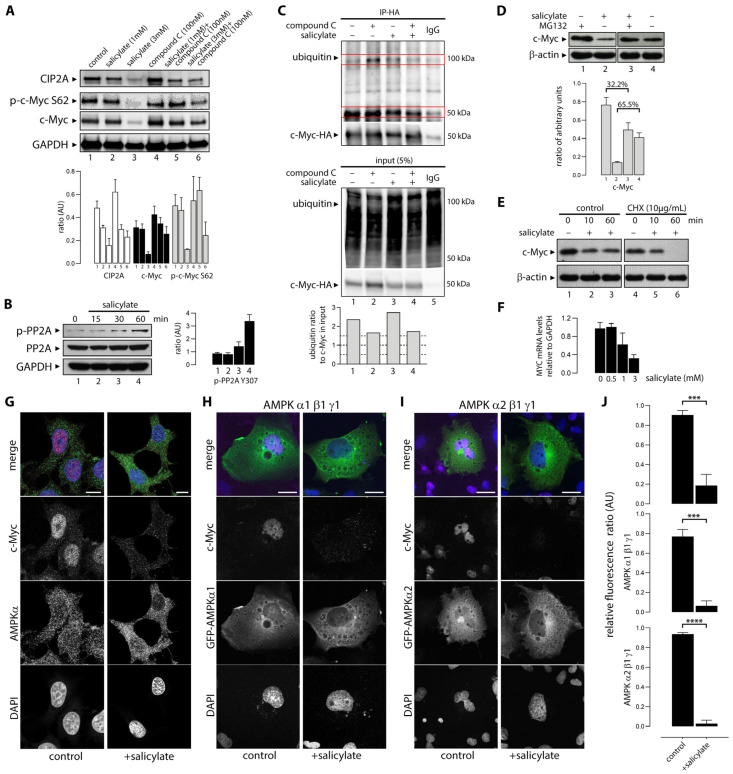
AMPK activation with sodium salicylate mediates c-Myc ubiquitinylation and nuclear depletion. (**A**) Exposure of HCT116 colon cancer cells to 1 or 3 mM salicylate for 48 h (lanes 2 and 3, respectively) elicited the loss of c-Myc expression accompanied by CIP2A, but no change in the levels of S62 phosphorylated c-Myc. Cells were also treated with a combination of salicylate (1 or 3 mM) and compound C (dorsomorphin 100 nM) for 8 h (lanes 5 and 6, respectively). (**B**) Salicylate induced phosphorylation of PP2A Y307. Samples were analyzed with an anti-pY307-PP2Ac antibody, and the blots were reprobed with an anti-PP2Ac antibody. (**C**) Sodium salicylate induced AMPK-mediated ubiquitinylation of c-Myc. HEK293 cells were transiently transfected with c-Myc-HA (lanes 1 through 4) or a mock construct (HA-vector, lane 5). Cells were then either left untreated (lanes 1 and 5) or treated with compound C (lane 2), sodium salicylate (lane 3), or a combination of both (lane 4), for three hours. C-Myc-HA-tagged protein was then immunopurified and its ubiquitination levels analyzed with a ubiquitin antibody by western blot (upper panel, ubiquitin). Immunoblots of the corresponding cell lysates (ubiquitin antibody) and purified proteins (HA antibody) are shown (lower panel). Red boxes highlight regions of interest corresponding to mono- and poly-ubiquitinylated c-Myc. (**D**,**E**) western blot detection of c-Myc in HCT116 cells treated with (**D**) MG-132 (20 μM) and (**E**) CHX (10 µg/mL) and in the presence or absence of 3 mM salicylate for 12 h. (**F**) QRT-PCR analysis of salicylate effects on relative levels of *MYC* mRNA in HCT116 cancer cells. *MYC* and *GAPDH* mRNA were quantified using a LightCycler Real-Time PCR system. Changes in mRNA levels of *MYC* were normalized to mRNA levels of *GAPDH*, and relative fold changes in mRNA levels were calculated compared to their respective vehicle-treated controls. Graphic bars indicate relative fold changes in mRNA ± SEM (arbitrary units) in each treatment group for each cell line. (**G**–**I**): sodium salicylate causes nuclear depletion of c-Myc. (**G**) AMPKα and c-Myc are visualized by indirect immunofluorescence in formaldehyde-fixed HCT116 cells. Also, COS cells were transfected with the GFP-tagged AMPK complex, either (**H**) α1β1γ1 or (**I**) α2β1γ1. Under normal growth conditions (control), c-Myc showed strong nuclear expression. Upon exposure to sodium salicylate (+salicylate), c-Myc was absent from the nuclear compartment. Scale bars: 10 µm. (**J**) Quantification of the nuclear signal of c-Myc relative to DAPI in HCT116 cells, and COS cells transfected with α1β1γ1 or α2β1γ1. *** *p* < 0.001; **** *p* < 0.0001.

One of the major regulatory mechanisms controlling c-Myc cellular protein levels is the targeted degradation of c-Myc through the ubiquitin/proteasome pathway [49]. This process is regulated through interdependent post-translational modifications able to stabilize and destabilize Myc. One of these modifications is the phosphorylation of S62, which enables a second phosphorylation of c-Myc at T58 that in turn licenses the protein for degradation [52]. Dephosphorylation of S62 by Protein Phosphatase 2A (PP2A) opposes the activation and degradation of Myc (illustrated in Figure 2B). PP2A is in turn regulated by Cancerous Inhibitor of PP2A (CIP2A), which also interacts directly with c-Myc (Figure 2B). We examined levels of c-Myc S62 phosphorylation in HCT116 cells upon exposure to salicylate alone (1 mM and 3 mM) and in combination with compound C (1 mM and 3 mM, respectively), but found no significant difference in S62 phosphorylation levels (Figure 5A). However, we observed downregulation of CIP2A in a dose-dependent manner (Figure 5A). Downregulation of CIP2A could only be partially reverted by compound C, suggestive of additional mechanisms to AMPK activation (Figure 5A, lanes 5 and 6). We also found that salicylate exposure caused increased phosphorylation of Tyr307 of PP2A without affecting the protein levels of PP2A (Figure 5B), suggesting salicylate inhibited PP2A enzymatic activity.

We then investigated changes in the ubiquitination levels of c-Myc upon exposure to salicylate. Recombinant HA-tagged c-Myc protein was transiently expressed in HEK293 cells. Cells were untreated or treated with salicylate, or compound C, or a combination of both (Figure 5C). Lysates were immunoprecipitated with an anti-HA antibody and the presence of c-Myc and protein ubiquitination examined by western blot analysis. Ubiquitinated bands were quantified, normalized to levels of c-Myc present in the lysate (input), and plotted (Figure 5C, lower panel). Salicylate induced an increase in the total protein ubiquitination levels (Figure 5C, input, ubiquitin panel) and a decrease in levels of c-Myc. Compound C treatment alone or in combination with salicylate decreased the total pool of ubiquitinylated proteins. Also, c-Myc protein level increased when cells were treated with compound C. Purified c-Myc-HA ubiquitination levels (Figure 5C, upper IP-HA panel) shows an accumulation of ubiquitinated c-Myc when basal AMPK activity was inhibited with compound C. Salicylate increased c-Myc ubiquitination when compared to the untreated sample. Concomitant AMPK inhibition and activation did not change c-Myc ubiquitin levels, as expected.

The end result of c-Myc ubiquitination would be a proteasome-mediated turnover of c-Myc. To determine the role of the proteosomal pathway in salicylate-induced degradation of c-Myc, we treated HCT116 cells with the peptide-aldehyde proteasome inhibitor MG132 (carbobenzoxyl-L-leucyl-L-leucyl-L-leucine) (Figure 5D). Cells were treated for 6 h with vehicle or 20 µM MG132, followed by another 8 h incubation with 3 mM salicylate or vehicle. MG132 partially prevented downregulation of c-Myc by salicylate. Salicylate reduced the level of c-Myc by 65.5% compared to the untreated control, but in the presence of MG132, this reduction was 32.2% (Figure 5D, lower panel), indicating that proteosomal degradation is partially responsible for the c-Myc protein downregulation caused by salicylate. To further explore the post-transcriptional mechanisms responsible for the decrease in c-Myc protein levels, we treated HCT116 cells with cycloheximide (CHX), a protein synthesis inhibitor. Blockage of protein synthesis with CHX exacerbated the reduction in c-Myc protein levels in HCT116 cells exposed to salicylate (Figure 5E). To determine whether exposure of HCT116 cells to salicylate affected c-Myc expression at the transcriptional level, we performed a qRT-PCR analysis of *MYC* mRNA levels in cells left untreated or treated with salicylate acid at 0.5, 1, and 3 mM concentrations for 24 h. As shown in Figure 5F, salicylate acid causes a significant decrease in *MYC* RNA levels in a dose-dependent fashion, at the 1 and 3 mM dose levels. These results show that the observed decrease in c-Myc protein levels caused by salicylate is due to effects at multiple levels, both transcriptional and post-translational.

### 3.7. Sodium Salicylate-Induced AMPK Activation Depleted Expression of Nuclear C-Myc

We demonstrated here that exposure to salicylate decreases cellular levels of the c-Myc protein. Myc is a short-lived nuclear protein, and post-translational modifications affect not only its stability and activity, but also its localization. Given that the cytoplasmic to nuclear distribution ratio of the c-Myc protein is regulated and functionally relevant [53,54], we investigated the effect of salicylate on c-Myc levels in the cytoplasm and nuclear compartments. Immunofluorescence analysis of HCT116 cells untreated or treated with 3 mM salicylate showed that loss of c-Myc occurred mainly in the nuclear form of the protein (Figure 5G,J).

Given that c-Myc depletion upon treatment with salicylate could be easily visualized by immunofluorescence, we used this assay to examine whether there was any isoform specificity to the salicylate-mediated downregulation of c-Myc. AMPK is a heterotrimeric complex consisting of the α catalytic subunit and two regulatory subunits (β and γ). Multiple isoforms of the catalytic (α1 and α2) and regulatory subunits (β1, β2, γ1, γ2, and γ3) have been identified [55]. AMPK complexes with α1 or α2 isoforms show tissue- and cell-specific expression patterns, suggesting that the two isoforms could play different physiological roles. Furthermore, the two isoforms of AMPKα, AMPKα1 and AMPKα2, can reportedly control tumor development in different ways [56]. Cells were transfected with plasmids encoding either AMPK α1β1γ1 subunits or α2β1γ1 subunits. Cells were left untreated or were treated for 1 h with 3 mM sodium salicylate. Figure 5G,H demonstrate that exposure of cells to salicylate significantly reduces c-Myc in cells with AMPK α1β1γ1 or α2β1γ1 subunits when compared to the untreated controls. This effect was observed independently of isoforms α1 or α2 (Figure 5H,I, respectively).

### 3.8. AMPK Activation Is Negatively Associated with C-Myc Expression in Colorectal Adenomas

Most, if not all, colorectal cancers arise through the adenoma–carcinoma sequence, a stepwise process where the colonic epithelium progresses from normal to adenoma to cancer. Multiple randomized clinical trials have demonstrated that aspirin is effective in preventing CRC disease also at early stages, reducing the risk of recurrent colorectal adenomas [57,58]. To determine whether AMPK activation was associated with c-Myc expression in precursor lesions in patients, we carried out a search for patients that had endoscopic removals of a high-grade adenomas and with no concurrent occurrence of cancer. We found that the expression of c-Myc in adenomas, evaluated by immunohistochemistry (illustrated in Figure 1E), was inversely correlated with the expression of p-AMPKα T172 (Figure 1F, *p* = 0.0418).

## 4. Discussion

The preventive effect of aspirin on colorectal cancer has been extensively studied and documented. However, this effect is only seen at daily doses of at least 75 mg and after a latency of, at best, 10 years [59], suggesting a protracted mechanism. Early investigations mainly focused on COX-dependent pathways related to inflammation [34]. But salicylate is pleiotropic and modulates several molecular targets, many of which may also contribute to its anticancer effect on CRC. To identify salicylate-induced changes associated with its effect on CRC, we compared the gene expression profiles of histologically normal colonic tissue samples collected from AOM-treated and AOM + salicylate-treated mice. The aspirin dosage we used in this study of ~60 mg/kg/day was based on previous work demonstrating that the effects observed in mice at this dose match those seen in humans on a medium-dose aspirin regimen (300 mg/daily) [60]. Conversion of this dosage, using known pharmacokinetic parameters (bioavailability F = 1; volume of distribution Vd = 0.2–0.3 L/kg; rapid absorption and elimination rates; and steady-state conditions from chronic dosing), was estimated to produce peak serum salicylate concentrations of approximately 1.6 mM. However, the local salicylate concentration in the gut lumen is expected to be somewhat higher, due to direct exposure during absorption and potential enterohepatic recirculation. Regular administration of salicylate in the AOM-induced mouse model of colon carcinogenesis reduced both colorectal tumor incidence and burden in treated animals (Figure 1), confirming the protective effect of salicylate. Transcriptomic analysis found one major common denominator, the proto-oncogene c-Myc, suggesting salicylate treatment affected the Myc-dependent transcriptional program in colonic cells. Immunohistochemical analysis of c-Myc confirmed its downregulation in salicylate-treated animals (Figure 1), and exposure of HCT116 colon cancer cells to salicylate caused downregulation of c-Myc, with a 65% decrease in protein abundance after 48 h exposure to 3 mM of salicylate (Figure 2A).

**Figure 6:**
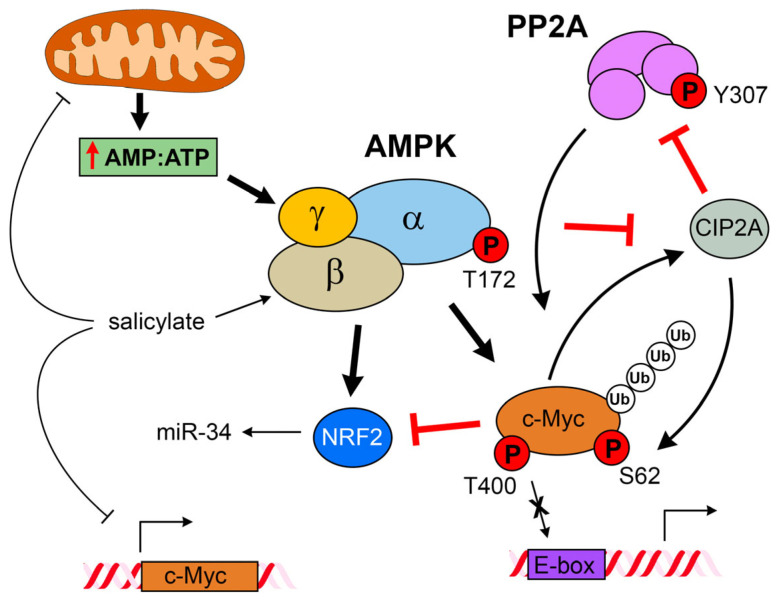
Model of potential mode of action of salicylate on c-Myc. Salicylate decreases the expression of *MYC* mRNA at the transcriptional level. In addition, salicylate activates AMPK both through directly binding to the ADaM site and changes in the mitochondrial membrane potential, which lead to an increased AMP:ATP ratio. Activated AMPK can directly bind to c-Myc and phosphorylate it at the bHLH-LZ region (S373 and T400), disrupting DNA binding. Additionally, phosphorylation of PP2A at Y307 and downregulation of CIP2A further compound the regulatory loop, decreasing c-Myc levels and affecting the c-Myc transcriptional program. Modulation of miR-34 defines another regulatory loop, further linking AMPK activation and c-Myc expression.

Previous studies have shown that salicylate activates AMPK, a key regulator of cellular energy homeostasis [20]. Other studies showed that AMPK activation with synthetic activators, such as metformin and phenformin, could also downregulate c-Myc levels in prostate and breast cancer cells [37,61,62]. Furthermore, aspirin and salicylic acid were also shown to decrease c-Myc expression in colon cancer cell lines [34]. These findings are substantiated in our study, in which we observed a causal relationship between the two processes, with salicylate exposure leading to concomitant AMPK activation and c-Myc downregulation (Figure 2A), through multiple direct and indirect mechanisms (Figure 6). As expected, activation of AMPK with synthetic activators, such as A76966 or phenformin, resulted in downregulation of c-Myc, paralleling the effect of salicylate (Figure 2B,C). Glucose starvation, a physiological stimulus, also caused downregulation of c-Myc (Figure 2D), showing AMPK activation induces c-Myc downregulation as a general effect. In all cases, blocking AMPK activation with compound C prevented the downregulation of c-Myc (Figure 2A–D), showing this effect to be associated to AMPK activation. Moreover, we showed that the synthetic activation of AMPK by exposure to salicylate or through glucose withdrawal induced AMPK/c-Myc association (Figure 4A–C), and that the AMPK-c-Myc protein complex brought about the phosphorylation of c-Myc (Figure 4D–F). In silico and in vitro analyses of AMPK-induced c-Myc phosphorylation identified several sites, of which two, Ser-373 and Thr-400, were predominantly affected (Figure 4G,H). These sites are located on the C-terminal basic helix–loop–helix leucine zipper (bHLH-LZ) region and can regulate Myc transcriptional activity [63]. This regulatory loop presumably underpins the effect of salicylate on the Myc-dependent transcriptional program we observed in the AOM + salicylate-treated mice.

Oncogenic c-Myc-driven cell growth and proliferation drain cellular energy resources, and the stress will activate AMPK [64]. There are multiple regulatory layers and checks maintaining the balance between the opposing cellular effects of AMPK and c-Myc. Our study identified a new direct interaction between the two protein complexes that closes the regulatory cycle, with activated AMPK binding c-Myc and phosphorylating it in the bHLH-LZ region, thus regulating its transcriptional activity (Figure 4). But beyond regulating the transcriptional potential of c-Myc itself, salicylate also causes decreased c-Myc protein abundance (Figure 2). One mechanism that controls Myc levels involves its phosphorylation-dependent proteolysis. The c-Myc protein has a very short half-life of 20 to 30 min in a regulated proteolytical process mediated by the ubiquitin–proteasome pathway. C-Myc’s half-life is strictly controlled by a phosphodegron, where simultaneous Thr-58 and Ser-62 phosphorylation serves as a ubiquitination signal and targets Myc for degradation. We could not see increased levels of c-Myc S62 phosphorylation upon salicylate exposure (Figure 5A), but since this is a transient modification that targets Myc for proteosome-mediated degradation, it could simply reflect the dynamic turnover of phosphorylated c-Myc. The AMPK-mediated downregulation of c-Myc seemed to follow a canonical path, as AMPK activation resulted in ubiquitination of c-Myc (Figure 5C) and quick depletion of nuclear c-Myc (Figure 5J). The reduction in c-Myc protein abundance appears to be partly due to a direct degradation of the c-Myc protein through proteolysis, as MG132 partially prevented downregulation of c-Myc by salicylate (Figure 5D) and CHX potentiated it (Figure 5E). But we also found that c-Myc gene transcription was affected by salicylate (Figure 5F), showing that salicylate impacts c-Myc protein abundance through multiple mechanisms. We could also identify an additional regulatory loop that was affected by salicylate, as we observed downregulation of CIP2A and phosphorylation of PP2A (Figure 5A,B, respectively). In a recent study looking to gain new insights into the molecular mechanisms underlying the chemopreventive effect of salicylate on CRC development, Liu and colleagues [65] uncovered yet another regulatory loop modulated by salicylate. Activation of AMPK caused activation of NRF2, which in turn directly induced miR-34a/b/c expression. Salicylate-mediated depletion of c-Myc completed the loop—Myc is a known repressor of NRF2, and depletion of c-MYC by salicylate was necessary for NRF2-mediated activation of miR-34a/b/c.

Expression of c-Myc is very strictly regulated, at multiple levels, in normal cells, but it is overexpressed and constantly activated in many cancers, including colon cancer, leading to poor prognosis and an aggressive metastatic phenotype [34,66]. Moreover, nuclear c-Myc overexpression can be an early event in carcinogenesis [67]. Chronic depletion of the c-Myc protein by AMPK activation would thus be expected to have a significant impact on cancer development: non-malignant cells can deal with declining ATP levels because AMPK regulates energy cellular pathways in response to metabolic alterations. This metabolic checkpoint mechanism arrests the cell cycle before the ATP levels drop too low, and thus allows cells time to recover their ATP reservoirs via catabolic pathways. On the other hand, cancer cells with deregulated growth signaling pathways driven by oncogenic drivers, such as *Ras*, which leads to deregulated c-Myc expression, often are unable to downregulate c-Myc, nor exit from the cell cycle, nor switch off the anabolic pathways. Therefore, these cells will continuously cycle under low ATP levels and with an unresponsive AMPK. In fact, cancer cells often show a progressive accumulation of c-Myc [68,69,70]. The findings in this study suggest that AMPK-activating compounds, some of which have been used for decades to treat metabolic disorders, can be useful for the treatment of cancer, and provide a mechanistic explanation for the cancer-preventing effects of AMPK-activating compounds [71,72,73]. Salicylate seems to have the added advantage of possessing a dual path to AMPK activation, as it can function as a direct activator of AMPK by allosteric activation and cause respiratory cellular stress. The injection of oligomycin A and subsequent inhibition of complex V revealed an increased proton leak (mitochondrial uncoupling) in a dose-dependent relation to salicylate (Figure 4D) [30,68]. Because of mitochondrial uncoupling, the respiration related to ATP production was reduced in the cells treated with salicylate. The maximum respiratory capacity was also lowered in a dose-dependent relation by the salicylate treatment, suggesting a compromised mitochondrial function. The resulting decreased ATP pool will lead to AMPK activation.

How do we explain the multilevel regulatory complexity elicited by salicylate revealed here? Salicylic acid has been used for medicinal purposes since ancient times. Using herbal extracts as a source of salicylic acid for the treatment of fever, joint pain, or inflammation has occurred as far back as 2500 BCE. Salicylic acid is a phytohormone with multiple physiological functions (reviewed in [74]). Our growing understanding of the role of salicylic acid in plants provides relevant insights into the effects of salicylate on human cells. (i) Improvement of seed germination: Salicylic acid improves seed germination by promoting the biosynthesis of several enzymes involved in metabolic pathways, such as the pentose phosphate pathway, glycolysis, and gluconeogenesis. This regulatory loop controlling metabolism counterparts what we saw with AMPK activation. (ii) Salicylic acid regulates the ubiquitin–proteasome system pathway as a mechanism to balance the antagonistic control of seed germination between the opposing plant hormones abscisic acid and gibberellins. (iii) Control of electron transport and oxidative phosphorylation in plant mitochondria: Salicylic acid inhibits both ATP synthesis and respiratory O_2_ uptake, possibly because both the uncoupling and inhibitory effects of salicylic acid in respiration would act to lower cell ATP levels in plants that accumulate salicylic acid, restricting their growth. (iv) Regulation of programmed cell death-associated genes and autophagy induction, involving multiple ATG genes. (v) Salicylic acid directly binds to A subunits of PP2A, inhibiting the activity of this complex in plants, which attenuates root growth [75]. (vi) Salicylic acid-mediated plant immune responses involve suppression of basic helix–loop–helix proteins MYC and MYB [76], and (vii) salicylic acid plays a critical role in regulating stress responses in plants, including defense against pathogens and abiotic stresses. While plants do not have NRF2, they possess antioxidant systems and stress-responsive transcription factors analogous to NRF2-dependent stress responses in animals. It is then hardly surprising that many of the physiological functions that salicylic acid has in plants are reiterated in the biochemical pathways that are conserved in humans.

## 5. Conclusions

Cells with increased c-Myc levels are aggressive, with high tumorigenic potential. Because c-Myc-driven cell growth and proliferation drain cellular energy resources and affect energy and redox homeostasis, the stress caused by these processes will engage the metabolic checkpoint kinase AMPK. There are multiple regulatory layers and checks maintaining the balance between the opposing cellular effects of AMPK and c-Myc. Our study identified a new, direct mechanistic interaction between the two protein complexes that closes the regulatory cycle, with activated AMPK binding c-Myc, phosphorylating it, and affecting its transcriptional program. Overall, our results suggest a dynamic role for AMPK in regulating c-Myc protein levels and function. Basal AMPK activity might be essential for c-Myc protein stability regulation. Our findings indicate that AMPK may be an essential regulator of the c-Myc-mediated transcriptional program in transformed colonic cancer whilst adapting to metabolic fluctuations during carcinogenesis, which would explain why the preventive effect of salicylate on CRC is protracted and requires chronic treatment with high levels. We suggest that the chronic depletion of c-Myc, brought about by salicylate-dependent AMPK activation, could account for the chemopreventive effect of aspirin on colorectal cancer, indicating a possible way forward for the therapeutic targeting of oncogenic Myc.

Salicylate has been shown to prevent both the onset of colorectal adenoma, a precancerous lesion, and CRC. Although chronic Myc depletion may prevent carcinogenesis, cells that have undergone malignant transformation could activate alternative oncogenic pathways that would bypass the need for Myc. Inactivation of miR-34a/b/c was shown to abrogate the inhibitory effects of salicylate on migration, invasion, and metastasis formation by CRC cells, providing a convincing molecular basis for the effect of salicylate both on premalignant lesions and cancers.

## Data Availability

Data reported in this paper will be shared by the lead contact upon request. This paper does not report original code.

## Supplementary Materials

The following supporting information can be downloaded at: https://www.mdpi.com/article/10.3390/cells14040294/s1, Table S1: Significance Analysis of Microarrays (SAM) identified 632 genes significantly deregulated by aspirin.
