# Supplementary material for: Salicylate-Elicited Activation of AMP-Activated Protein Kinase Directly Triggers Degradation of C-Myc in Colorectal Cancer Cells
Source: Cells. 2025 Feb 17;14(4):294. doi: 10.3390/cells14040294 (PMC11854256; doi:10.3390/cells14040294)
Supplement: Supplementary file 1 [file cells-14-00294-s001.zip › cells-3197796-supplementary.pdf]

Table S1. Significance Analysis of Microarrays (SAM) identified 632 genes significantly deregulated by aspirin.

	GB_ACC_GB_ACC	GENE_SYMBOL_(upper)	GENE_NAME_GENE_NAME	Fold change	q-value (%)
1	NM_011352	SEMA7A	sema domain, immunoglobulin domain (Ig), and GPI membrane anchor, (semaphorin) 7A	0,15749349	0
2	NM_145996	ARID5A	AT rich interactive domain 5A (Mrf1 like)	0,09954637	0
3	NM_009642	AGTRAP	angiotensin II, type I receptor-associated protein	0,16890587	0
4	NM_009795	CAPNS1	calpain, small subunit 1	0,1679081	0
5	XM_991213	OTUD1	OTU domain containing 1	0,12009008	0
6	XM_910141	LOC635169	hypothetical LOC635169	0,15549704	0
7	NM_010556	IL3	interleukin 3	0,25485113	0
8	NM_010956	OGDH	oxoglutarate dehydrogenase (lipoamide)	0,19761969	0
9	NM_031172	TRIM17	tripartite motif protein 17	0,2061697	0
10	NM_144810	KLHDC8A	kelch domain containing 8A	0,09270379	0
11	NM_029998	6030458C11RIK	RIKEN cDNA 6030458C11 gene	0,0865363	0
12	NM_029061	CCDC7	coiled-coil domain containing 7	0,20901605	0
13	NM_028283	UACA	uveal autoantigen with coiled-coil domains and ankyrin repeats	0,03727608	0
14	NM_025367	SPHK1	sphingosine kinase 1	0,15114929	0
15	BC054536	ADAM11	a disintegrin and metallopeptidase domain 11	0,28481224	0
16	NM_010607	KCNK2	potassium channel, subfamily K, member 2	0,11672657	0
17	NM_009681	AP3S1	adaptor-related protein complex 3, sigma 1 subunit	0,2619507	0
18	NM_010256	GART	phosphoribosylglycinamide formyltransferase	0,14860097	0
19	NM_173861	CSNKA2IP	casein kinase 2, alpha prime interacting	0,17441152	0

			protein(Csnka2ip)		
20	XM_907275	LOC632964	similar to basic transcription factor 3	0,23245361	0
21	XM_913724	LOC637908	similar to putative pheromone receptor (Go-VN5)	0,20826338	0
22	AK086918	A430090L17RIK	RIKEN cDNA A430090L17 gene	0,21888591	0
23	NM_009023	RAPSN	receptor-associated protein of the synapse	0,3157084	0
24	AK008890	ZFP84	zinc finger protein 84(Zfp84)	0,08505253	0
25	AK043729	IARS2	isoleucine-tRNA synthetase 2, mitochondrial	0,2323636	0
26	AK078133	SENP5	SUMO/sentrin specific peptidase 5(Senp5)	0,20526204	0
27	NM_027652	EPT1	ethanolaminephosphotransferase 1 (CDP-ethanolamine-specific)(Ept1)	0,26484144	0
28	AK078120	PTP4A1	protein tyrosine phosphatase 4a1	0,06613237	0
29	AK042173	CYTIP	cytohesin 1 interacting protein(Cytip)	0,16327526	0
30	BC005693	HMGN3	high mobility group nucleosomal binding domain 3	0,07946318	0
31	NM_007942	EPO	erythropoietin	0,15346876	0
32	NM_001033367	NLRC4	NLR family, CARD domain containing 4(Nlrc4)	0,2604705	0
33	NM_007937	EPHA5	Eph receptor A5	0,16954848	0
34	NM_025864	TMEM206	transmembrane protein 206(Tmem206)	0,2310525	0
35	AK009124	2310002L09RIK	RIKEN cDNA 2310002L09 gene	0,09260914	0
36	NM_177198	4833432P19RIK	RIKEN cDNA 4833432P19 gene	0,3503101	0
37	BC080815	KNL1	kinetochore scaffold 1(Knl1)	0,29889485	0
38	NM_026147	RPS20	ribosomal protein S20	0,21029381	1,4785321
39	NM_173011	IDH2	isocitrate dehydrogenase 2 (NADP+), mitochondrial	0,26528016	1,4785321

40	NM_007808	CYCS	cytochrome c, somatic	0,13464165	1,4785321
41	NM_178873	ADCK2	aarF domain containing kinase 2	0,23414557	1,4785321
42	NM_080793	SETD7	SET domain containing (lysine methyltransferase) 7	0,11323115	1,4785321
43	BC066073	ZFP800	zinc finger protein 800(Zfp800)	0,15858859	1,4785321
44	NM_018744	SEMA6A	sema domain, transmembrane domain (TM), and cytoplasmic domain, (semaphorin) 6A	0,26933447	1,4785321
45	NM_028660	KLK9	kallikrein 9	0,24959603	1,4785321
46	NM_010783	MDFI	MyoD family inhibitor	0,17650026	1,4785321
47	AK083542	TBX3OS2	T-box 3, opposite strand 2(Tbx3os2)	0,12544063	1,4785321
48	NM_177764	VMN2R57	vomeronasal 2, receptor 57(Vmn2r57)	0,20477124	1,4785321
49	AK034148	CASC1	cancer susceptibility candidate 1	0,2678749	1,4785321
50	BC021611	ACSF3	acyl-CoA synthetase family member 3(Acsf3)	0,16902694	1,4785321
51	AK050937	FAM169A	family with sequence similarity 169, member A(Fam169a)	0,22829658	1,4785321
52	NM_177768	ZBTB44	zinc finger and BTB domain containing 44(Zbtb44)	0,20541513	1,4785321
53	NM_027251	2010107G23RIK	RIKEN cDNA 2010107G23 gene	0,13560878	1,4506353
54	AK158895	PRRT2	proline-rich transmembrane protein 2(Prrt2)	0,18170492	1,4237716
55	AK147576	PRICKLE2	prickle-like 2 (Drosophila)	0,3855137	1,3729227
56	BC087897	AGBL3	ATP/GTP binding protein-like 3	0,23071174	1,3729227
57	NM_019694	LETM1	leucine zipper-EF-hand containing transmembrane protein 1	0,30074885	1,3031131
58	NM_145525	OSBPL6	oxysterol binding protein-like 6	0,25590938	1,3031131
59	NM_026724	RPL34	ribosomal protein L34(Rpl34)	0,28166163	1,3031131

60	NM_018804	SYT11	synaptotagmin XI	0,12751958	1,2813945
61	NM_011804	CREG1	cellular repressor of E1A-stimulated genes 1	0,24998595	1,260388
62	NM_013742	CARS	cysteinyl-tRNA synthetase	0,17855701	1,2013073
63	AF168769	NPAS3	neuronal PAS domain protein 3	0,30042428	1,2013073
64	NM_013685	TCF4	transcription factor 4	0,33693013	1,2013073
65	AK131718	IPP	IAP promoted placental gene	0,2709553	1,1828257
66	NM_026359	4930578I06RIK	RIKEN cDNA 4930578I06 gene	0,27530473	1,1649041
67	AK122427	TTC28	tetratricopeptide repeat domain 28	0,19527352	1,1475174
68	AK008872	4930404N11RIK	RIKEN cDNA 4930404N11 gene	0,2095983	1,1142561
69	NM_144862	LIMS2	LIM and senescent cell antigen like domains 2	0,20369102	1,1142561
70	NM_146792	OLFR1246	olfactory receptor 1246	0,15496784	1,0983381
71	NM_027896	COASY	Coenzyme A synthase	0,1407857	1,0389686
72	AK015079	4930404H11RIK	RIKEN cDNA 4930404H11 gene	0,15650155	1,0389686
73	NM_008473	KRT1	keratin 1(Krt1)	0,27250063	1,0389686
74	NM_172258	SLC36A3	solute carrier family 36 (proton/amino acid symporter), member 3	0,2464026	1,0389686
75	AF150755	MACF1	microtubule-actin crosslinking factor 1	0,23247884	1,0116272
76	NM_029437	CKAP5	cytoskeleton associated protein 5	0,20985731	1,0116272
77	AK007164	PRR30	proline rich 30(Prr30)	0,17538054	0,9856881
78	NM_019402	PABPN1	poly(A) binding protein, nuclear 1	0,3216685	0,9856881
79	AK147474	MTAP1A	microtubule-associated protein 1 A	0,2862293	0,973211
80	NM_134194	VMN1R225	vomeronasal 1 receptor 225(Vmn1r225)	0,45313787	0,96104586
81	AK077967	PAK7	p21 (CDKN1A)-activated kinase 7	0,17507176	0,93760574
82	NM_007396	ACVR2A	activin receptor IIA	0,3754616	0,93760574
83	BC094568	TAF1	TAF1 RNA polymerase II, TATA box binding protein	0,22650863	0,9263093

			(TBP)-associated factor		
84	AK080318	FMNL2	formin-like 2	0,28382674	0,9152818
85	AK002842	0610039K10RIK	RIKEN cDNA 0610039K10 gene	0,25319487	0,8939962
86	AK040949	NEURL3	neuralized E3 ubiquitin protein ligase 3(Neurl3)	0,2790677	0,8939962
87	NM_025901	POLR3K	polymerase (RNA) III (DNA directed) polypeptide K	0,20765123	0,86386144
88	XM_129836	PHF3	PHD finger protein 3	0,29753873	0,86386144
89	NM_026230	H2AFY3	H2A histone family, member Y3	0,23606704	0,86386144
90	AK144901	UBALD2	UBA-like domain containing 2(Ubald2)	0,16029017	0,854263
91	NM_172743	PLEKHA7	pleckstrin homology domain containing, family A member 7	0,12956418	0,8448755
92	NM_026282	SPBC24	spindle pole body component 24 homolog (S. cerevisiae)	0,26453623	0,80087155
93	NM_175258	RAPGEF6	Rap guanine nucleotide exchange factor (GEF) 6	0,2256821	0,80087155
94	NM_008983	PTPRK	protein tyrosine phosphatase, receptor type, K	0,23250042	0,80087155
95	BC012016	PRDM9	VPR domain containing 9	0,21036051	0,80087155
96	NM_021415	CACNA1H	calcium channel, voltage-dependent, T type, alpha 1H subunit	0,32713178	0,80087155
97	NM_028112	SEH1L	SEH1-like (S. cerevisiae	0,23351993	1,5852304
98	AK148962	GAK	cyclin G associated kinase	0,12041993	1,5532055
99	AK048567	NRCAM	neuronal cell adhesion molecule(Nrcam)	0,25657922	1,5532055
100	AK161694	AW495713	expressed sequence AW495713	0,26351464	1,5224489
101	NM_172930	TMEM255A	transmembrane protein 255A(Tmem255a)	0,20052412	1,5224489
102	NM_008858	PRKD1	protein kinase D1(Prkd1)	0,23045531	1,4785321
103	NM_177765	TTLL13	tubulin tyrosine ligase-like family,	0,1514966	1,4785321

			member 13(Ttll13)		
104	NM_010249	GABPB1	GA repeat binding protein, beta 1	0,24567628	1,4785321
105	AK011730	ZFP715	zinc finger protein 715	0,43552637	1,437078
106	NM_146317	OLFR725	olfactory receptor 725	0,37195635	1,437078
107	AK162429	SLC38A1	solute carrier family 38, member 1	0,18808027	1,437078
108	NM_020584	TERF2IP	telomeric repeat binding factor 2, interacting protein	0,28628913	1,4237716
109	XM_139711	ARID1B	AT rich interactive domain 1B (Swi1 like)	0,43227154	1,4107095
110	NM_024448	RAB12	RAB12, member RAS oncogene family	0,08590727	1,397885
111	NM_027629	PGM2L1	phosphoglucomutase 2-like 1	0,28488415	1,3852913
112	AK049415	AU020206	expressed sequence AU020206	0,17158437	1,360773
113	NM_013903	MMP20	matrix metallopeptidase 20 (enamelysin)	0,39970157	1,360773
114	NM_009486	VMN2R89	vomeronasal 2, receptor 89(Vmn2r89)	0,3369261	1,6428134
115	AK133210	4930544L04RIK	RIKEN cDNA 4930544L04 gene	0,19332892	1,6428134
116	NM_026301	RNF125	ring finger protein 125	0,34275934	1,6428134
117	NM_177281	D930030D11RIK	RIKEN cDNA D930030D11 gene	0,2519869	1,6428134
118	XM_619530	JMJD1B	jumonji domain containing 1B	0,39948928	1,9546696
119	NM_175308	MOBKL2C	MOB1, Mps One Binder kinase activator-like 2C (yeast)	0,28317717	1,9220917
120	AK078849	MPP7	membrane protein, palmitoylated 7 (MAGUK p55 subfamily member 7)	0,25453115	1,9220917
121	NM_144533	NMNAT3	nicotinamide nucleotide adenylyltransferase 3	0,26910955	1,9062067
122	NM_009700	AQP4	aquaporin 4	0,38429615	1,8752115
123	BC053027	MAPK8	mitogen activated protein kinase 8	0,23349983	1,8752115
124	NM_009450	TUBB2A	tubulin, beta 2a	0,2650036	1,8600888

125	NM_008811	PDHA2	pyruvate dehydrogenase E1 alpha 2	0,31770724	1,8305635
126	NM_175273	FAM219B	family with sequence similarity 219, member B(Fam219b)	0,22037514	1,8305635
127	NM_001011857	OLFR685	olfactory receptor 685	0,19713981	1,9786239
128	NM_183113	4932414N04RIK	RIKEN cDNA 4932414N04 gene	0,3521924	1,9786239
129	NM_133684	MARC2	MOCO sulphurase C-terminal domain containing 2	0,3067983	1,9786239
130	NM_025890	KHDC3	KH domain containing 3, subcortical maternal complex member(Khdc3)	0,4626675	1,9786239
131	NM_026616	RNASEH2C	ribonuclease H2, subunit C(Rnaseh2c)	0,17905886	1,9786239
132	NM_028634	CBY1	chibby homolog 1 (Drosophila)(Cby 1)	0,39464685	1,9786239
133	AK129282	SPAST	spastin	0,18376586	1,9786239
134	NM_008803	PDE8A	phosphodiestera se 8A	0,2485972	1,9786239
135	NM_011734	SIAE	sialic acid acetylesterase	0,28466466	1,9786239
136	AK053145	SLIT2	slit homolog 2 (Drosophila)	0,2604867	1,9786239
137	NM_013655	CXCL12	chemokine (C-X-C motif) ligand 12	0,39217955	2,181097
138	NM_020048	MED20	mediator complex subunit 20(Med20)	0,40551758	2,181097
139	NM_011306	RXRB	retinoid X receptor beta	0,2543544	2,181097
140	XM_126489	FOXK2	forkhead box K2	0,4875957	2,181097
141	NM_026820	IFITM1	interferon induced transmembrane protein 1	0,16337219	2,181097
142	NM_001013380	DYNC1LI2	dynein, cytoplasmic 1 light intermediate chain 2	0,33571526	2,1209288
143	NM_010442	HMOX1	heme oxygenase (decycling) 1	0,20342007	2,1209288
144	NM_177838	FAM163A	family with sequence similarity 163, member A(Fam163a)	0,13909987	2,1209288
145	NM_010132	EMX2	empty spiracles homolog 2 (Drosophila)	0,29922011	2,1209288

146	NM_144815	CECR5	cat eye syndrome chromosome region, candidate 5 homolog (human)	0,27672338	1,9713762
147	AK031736	TECPR2	tectonin beta-propeller repeat containing 2(Tecpr2)	0,21882768	1,9713762
148	AK051321	D030068K23RIK	RIKEN cDNA D030068K23 gene	0,35346144	1,9713762
149	NM_001007580	GM784	gene model 784, (NCBI)	0,25408202	1,9713762
150	AK031449	BC037438	cDNA sequence BC037438	0,44336313	1,9713762
151	AK019647	4930478K11RIK	RIKEN cDNA 4930478K11 gene	0,29421338	1,9713762
152	XM_001000244	4922502B01RIK	RIKEN cDNA 4922502B01 gene	0,4597286	1,9713762
153	NM_027865	TMEM25	transmembrane protein 25	0,21805641	1,9713762
154	AK039302	D230040A04RIK	RIKEN cDNA D230040A04 gene	0,39419198	1,9713762
155	NM_147037	OLFR1413	olfactory receptor 1413	0,24594003	1,9713762
156	NM_173415	NYX	nyctalopin	0,41297176	1,9713762
157	BC059729	NDUFA11	NADH dehydrogenase (ubiquinone) 1 alpha subcomplex 11	0,4420963	1,946422
158	NM_028735	TTC21A	tetratricopeptide repeat domain 21A	0,17413557	1,946422
159	NM_028148	SCAF11	SR-related CTD-associated factor 11(Scaf11)	0,36933774	1,9341804
160	NM_011262	DPF2	D4, zinc and double PHD fingers family 2	0,21955849	1,8983623
161	NM_144913	MEPCE	methylphosphat e capping enzyme(Mepce)	0,26309198	1,8983623
162	NM_026217	ATG12	autophagy-related 12 (yeast)	0,2625355	1,8983623
163	NM_020509	RETNLA	resistin like alpha	0,24595606	1,8867159
164	NM_013872	PMM1	phosphomanno mutase 1	0,25469962	1,8415251
165	NM_194059	NANOS3	nanos homolog 3 (Drosophila)	0,17308076	1,8415251
166	NM_138589	UBFD1	ubiquitin family domain containing 1(Ubfd1)	0,2467223	1,8415251
167	NM_010329	PDPN	podoplanin	0,4538013	1,8415251

168	AK085551	9230110F11RIK	RIKEN cDNA 9230110F11 gene	0,33812967	1,8090276
169	AK035528	9530053A07RIK	RIKEN cDNA 9530053A07 gene	0,20934172	1,8090276
170	NM_147201	NRBP1	nuclear receptor binding protein 1(Nrbp1)	0,35337424	1,8090276
171	NM_139228	RHBDL3	rhomboid, veinlet-like 3 (Drosophila)	0,31829977	1,7984484
172	NM_178869	TTLL1	tubulin tyrosine ligase-like 1	0,20072098	1,757341
173	NM_026504	COQ5	coenzyme Q5 homolog, methyltransferase (yeast)	0,36694938	1,757341
174	NM_146676	OLFR826	olfactory receptor 826	0,322853	1,757341
175	NM_027085	CLIC3	chloride intracellular channel 3	0,42272222	1,757341
176	NM_153419	GRWD1	glutamate-rich WD repeat containing 1	0,30674762	1,7473562
177	NM_007592	CAR8	carbonic anhydrase 8	0,20969078	1,9546696
178	NM_026331	SLC25A37	solute carrier family 25, member 37	0,36608	1,9220917
179	AJ491325	RP1HL1	retinitis pigmentosa 1 homolog (human)-like 1	0,20368329	1,9220917
180	NM_027290	MCM10	minichromosome maintenance deficient 10 (S. cerevisiae)	0,27999553	1,9220917
181	NM_008529	LY6E	lymphocyte antigen 6 complex, locus E	0,4719179	2,1121888
182	NM_019764	AMOTL2	angiomotin like 2	0,39185372	2,1121888
183	NM_026046	ZFP329	zinc finger protein 329	0,41628718	2,08923
184	AK087199	PPP1R16B	protein phosphatase 1, regulatory (inhibitor) subunit 16B	0,232351	2,08923
185	NM_001037926	ZFP987	zinc finger protein 987(Zfp987)	0,26111603	2,0667653
186	AK044788	HECTD1	HECT domain containing 1	0,2957661	2,0667653
187	NM_017392	CELSR2	cadherin EGF LAG seven-pass G-type receptor 2	0,3947145	2,0557132
188	AF148511	RBPMS	RNA binding protein gene with multiple splicing	0,19706008	2,0232544
189	NM_054073	TSGA13	testis specific gene A13	0,25281876	2,0232544

190	AK020241	9030201C23RIK	RIKEN cDNA 9030201C23 gene	0,39871958	2,0232544
191	AK084295	THAP6	THAP domain containing 6	0,23497109	2,0126615
192	AK082693	C230088H06RIK	RIKEN cDNA C230088H06 gene	0,4994675	2,002179
193	NM_026483	MPHOSPH10	M-phase phosphoprotein 10 (U3 small nucleolar ribonucleoprotein)	0,3476209	1,9415069
194	NM_007828	DAPK3	death-associated kinase 3	0,3140729	1,9415069
195	NM_178066	TMEM198B	transmembrane protein 198b(Tmem198b)	0,25919366	1,9415069
196	NM_080858	ASB12	ankyrin repeat and SOCS box-containing protein 12	0,14721686	1,9415069
197	NM_010251	GABRA4	gamma-aminobutyric acid (GABA-A) receptor, subunit alpha 4	0,41970867	1,9415069
198	NM_011705	VRK1	vaccinia related kinase 1	0,3650156	1,9415069
199	NM_028696	NABP1	nucleic acid binding protein 1(Nabp1)	0,42735577	2,295035
200	NM_030221	NADSYN1	NAD synthetase 1	0,29335514	2,295035
201	NM_001039094	NEGR1	neuronal growth regulator 1	0,17775184	2,295035
202	NM_008822	PEX7	peroxisome biogenesis factor 7	0,2519769	2,2612844
203	AK031605	TTC14	tetratricopeptide repeat domain 14	0,19458038	2,2612844
204	NM_011056	PDE4D	phosphodiesterase 4D, cAMP specific	0,26402935	2,2612844
205	NM_175392	MIGA2	mitoguardin 2(Miga2)	0,19021042	2,2393303
206	AK053308	8030475D13RIK	RIKEN cDNA 8030475D13 gene	0,36673328	2,2393303
207	XM_486124	BC033932	cDNA sequence BC033932	0,42376432	2,175953
208	NM_175515	INTU	inturned planar cell polarity protein(Intu)	0,30479398	2,175953
209	NM_028973	LRRC15	leucine rich repeat containing 15	0,37758493	2,175953
210	NM_175410	A930002I21RIK	RIKEN cDNA A930002I21 gene	0,30805105	2,175953
211	NM_009681	AP3S1	adaptor-related protein complex	0,40852863	2,175953

			3, sigma 1 subunit		
212	BC019785	HYOU1	hypoxia up-regulated 1	0,19638261	2,175953
213	NM_008550	MAN2B2	mannosidase 2, alpha B2	0,19805326	2,1356575
214	NM_144905	TMEM268	transmembrane protein 268(Tmem268)	0,30256838	2,1356575
215	AK039679	C130071C03RIK	RIKEN cDNA C130071C03 gene(C130071C03Rik)	0,37982205	2,1356575
216	NM_053096	NAT8F2	N-acetyltransferase 8 (GCN5-related) family member 2(Nat8f2)	0,36315694	2,1356575
217	XM_132143	SRP72	signal recognition particle 72	0,2946578	2,302967
218	AK017825	OIP5	Opa interacting protein 5	0,30808324	2,3399377
219	NM_146824	OLFR273	olfactory receptor 273	0,39017463	2,3399377
220	AK015681	CHIC2	cysteine-rich hydrophobic domain 2	0,4561245	2,3399377
221	NM_015765	HSPA14	heat shock protein 14	0,3113751	2,3399377
222	NM_145404	PRMT7	protein arginine N-methyltransferase 7	0,3730549	2,3399377
223	NM_009635	AVIL	advillin	0,3594148	2,3399377
224	NM_178397	FAF2	Fas associated factor family member 2(Faf2)	0,4531411	2,3399377
225	BC052065	RAP1GAP	Rap1 GTPase-activating protein	0,27087614	2,3399377
226	XR_003818	LOC672341	similar to 60S ribosomal protein L21	0,14394814	2,3399377
227	AK019612	4930445E18RIK	RIKEN cDNA 4930445E18 gene	0,4239942	2,3399377
228	NM_030172	EFCAB11	EF-hand calcium binding domain 11(Efcab11)	0,3343578	2,3399377
229	AK140530	PLCL1	phospholipase C-like 1	0,31190634	2,3399377
230	NM_029987	RPE65	retinal pigment epithelium 65	0,4789715	2,3399377
231	AK122234	RAPGEF5	Rap guanine nucleotide exchange factor (GEF) 5	0,32387194	2,3298082
232	NM_146174	TCAF2	TRPM8 channel-associated factor 2(Tcaf2)	0,49036932	2,30981
233	NM_053124	SMARCA5	SWI/SNF related, matrix associated, actin dependent	0,28407726	2,30981

			regulator of chromatin, subfamily a, member 5		
234	XR_001708	LOC665242	similar to Mucin-2 precursor (Intestinal mucin 2)	0,3588562	2,299939
235	NM_028136	DHX36	DEAH (Asp-Glu-Ala-His) box polypeptide 36	0,4052564	2,4330275
236	NM_026163	PKP2	plakophilin 2	0,19958399	2,4330275
237	AK133372	RFX8	regulatory factor X 8(Rfx8)	0,43874085	2,4330275
238	BC021773	GLB1L	galactosidase, beta 1-like	0,17509398	2,5311496
239	NM_133791	WWC2	WW, C2 and coiled-coil domain containing 2	0,3436679	2,5311496
240	NM_010630	KIFC2	kinesin family member C2	0,26494893	2,5311496
241	NM_011414	SLPI	secretory leukocyte peptidase inhibitor	0,5037031	2,5311496
242	AK054255	PXDN	peroxidasin homolog (Drosophila)	0,41662765	2,5311496
243	AK134636	2610005L07RIK	RIKEN cDNA 2610005L07 gene	0,11395582	2,5311496
244	NM_134180	V1RC25	vomeronasal 1 receptor, C25	0,30230612	2,4602776
245	BC049570	ATP8B3	ATPase, Class I, type 8B, member 3	0,36483037	2,4602776
246	NM_177678	ABLIM2	actin-binding LIM protein 2	0,33167207	2,4602776
247	XM_001002308	4833417A11RIK	RIKEN cDNA 4833417A11 gene	0,28777334	2,4602776
248	NM_010657	HIVEP3	human immunodeficiency virus type I enhancer binding protein 3	0,24369912	2,4602776
249	NM_153157	OLFM3	olfactomedin 3	0,24613887	2,4602776
250	NM_201255	KRT9	keratin 9	0,43001407	2,4602776
251	BC099569	1700012P22RIK	RIKEN cDNA 1700012P22 gene	0,25770065	2,5728788
252	NM_146052	LRRC3B	leucine rich repeat containing 3B	0,22203657	2,5728788
253	NM_019442	STK19	serine/threonine kinase 19	0,258928	2,5728788
254	NM_008649	MUP5	major urinary protein 5	0,39906526	2,5728788
255	NM_016927	PKD2L2	polycystic kidney disease 2-like 2	0,5452394	2,542845
256	NM_001017985	C2CD3	C2 calcium-dependent domain	0,29633376	2,542845

			containing 3(C2cd3)		
257	NM_011306	RXRB	retinoid X receptor beta	0,28490075	2,542845
258	NM_008840	PIK3CD	phosphatidylinositol 3-kinase catalytic delta polypeptide	0,41920656	2,5232093
259	AK034547	GHR	growth hormone receptor	0,25961566	2,5232093
260	AK051137	PTGER4	prostaglandin E receptor 4 (subtype EP4)	0,3523881	2,5038743
261	NM_007481	ARF6	ADP-ribosylation factor 6	0,34469032	2,5038743
262	NM_001039484	KCNJ10	potassium inwardly-rectifying channel, subfamily J, member 10	0,4946287	2,4943175
263	NM_001003909	ANKIB1	ankyrin repeat and IBR domain containing 1	0,28041062	2,46608
264	AK003565	1110008E08RIK	RIKEN cDNA 1110008E08 gene	0,312279	2,46608
265	NM_008717	ZFP638	zinc finger protein 638(Zfp638)	0,4851783	2,46608
266	AK142280	RAD23B	RAD23b homolog (S. cerevisiae)	0,41404155	2,456809
267	NM_134010	NUP107	nucleoporin 107	0,33978146	2,4384747
268	NM_016668	BHMT	betaine-homocysteine methyltransferase	0,48826873	2,4384747
269	AK016926	PRKAG2OS1	protein kinase, AMP-activated, gamma 2 non-catalytic subunit, opposite strand 1(Prkag2os1)	0,46377015	2,4294097
270	NM_026643	CEP57L1	centrosomal protein 57-like 1(Cep57l1)	0,2998179	2,3764043
271	NM_178367	DHX33	DEAH (Asp-Glu-Ala-His) box polypeptide 33	0,50741017	2,3764043
272	NM_013886	HDGFRP3	hepatoma-derived growth factor, related protein 3	0,3854345	2,3764043
273	NM_008030	FMO3	flavin containing monooxygenase 3	0,32682887	2,3764043
274	NM_011346	SELL	selectin, lymphocyte	0,26583138	2,3764043
275	AK013604	2900027M19RIK	RIKEN cDNA 2900027M19 gene	0,17564522	2,3764043
276	AK054366	ZMIZ1	zinc finger, MIZ-type containing 1(Zmiz1)	0,30217755	2,5992699

277	NM_026411	1700021F05RIK	RIKEN cDNA 1700021F05 gene	0,49481544	2,5992699
278	NM_021445	CTS6	cathepsin 6	0,4409452	2,5992699
279	NM_011136	POU2AF1	POU domain, class 2, associating factor 1	0,36626834	2,5992699
280	AK019535	IQCH	IQ motif containing H	0,2584088	2,5992699
281	NM_001017955	ZSCAN18	zinc finger and SCAN domain containing 18(Zscan18)	0,57348067	2,5992699
282	NM_009189	SIX1	sine oculis-related homeobox 1 homolog (Drosophila)	0,5069651	2,5360932
283	NM_016845	ACRBP	proacrosin binding protein	0,34382737	2,5360932
284	NM_178725	LRRC4C	leucine rich repeat containing 4C	0,26420063	2,5360932
285	NM_009038	RCVRN	recoverin	0,2853996	2,5360932
286	NM_024263	MXRA8	matrix-remodelling associated 8	0,3478213	2,5360932
287	XM_895070	TAOK1	TAO kinase 1	0,2131479	2,5360932
288	BC034843	ADAMTSL5	ADAMTS-like 5	0,3406031	2,5360932
289	AK137588	WWP1	WW domain containing E3 ubiquitin protein ligase 1	0,25439858	2,6420505
290	NM_009113	S100A13	S100 calcium binding protein A13	0,41687065	2,6420505
291	NM_175454	HID1	HID1 domain containing(Hid1)	0,50583386	2,6420505
292	NM_172917	E330039K12RIK	RIKEN cDNA E330039K12 gene	0,49553794	2,6062262
293	AK017692	JAM3	junction adhesion molecule 3	0,34991512	2,6062262
294	AK037771	UBR1	ubiquitin protein ligase E3 component n-recognin 1	0,25773528	2,6062262
295	AK132449	CAPN8	calpain 8	0,34819955	2,6062262
296	NM_008159	GPR33	G protein-coupled receptor 33	0,2580513	2,5886757
297	NM_025909	OMA1	OMA1 homolog, zinc metallopeptidase (S. cerevisiae)	0,3319279	2,5886757
298	AK044661	ACER3	alkaline ceramidase 3(Acer3)	0,3429851	2,579989
299	NM_175494	ZFP367	zinc finger protein 367	0,48673138	2,5542748

300	AK006067	1700017I07RIK	RIKEN cDNA 1700017I07 gene	0,31927034	2,5542748
301	NM_011180	CYTH1	cytohesin 1(Cyth1)	0,21578284	2,5542748
302	AK037393	LOC545785	hypothetical protein LOC545785	0,32130858	2,4801183
303	NM_008006	FGF2	fibroblast growth factor 2	0,2490154	2,4801183
304	NM_183038	DEFB39	defensin beta 39	0,45563185	2,4801183
305	NM_001039579	PPP4R1L-PS	protein phosphatase 4, regulatory subunit 1-like, pseudogene(Ppp4r1l-ps)	0,16319722	2,4801183
306	BC034876	1110008L16RIK	RIKEN cDNA 1110008L16 gene	0,2536558	2,4801183
307	BC098072	MORC3	microrchidia 3	0,37107465	2,4801183
308	NM_028711	SLC25A27	solute carrier family 25, member 27	0,4150034	2,4801183
309	NM_007765	CRMP1	collapsin response mediator protein 1	0,29067862	2,4801183
310	NM_031367	H28	histocompatibility 28	0,4825501	2,4801183
311	NM_031247	GIMAP3	GTPase, IMAP family member 3	0,25219843	2,4721437
312	NM_019808	PDLIM5	PDZ and LIM domain 5	0,44685298	2,4563472
313	NM_011212	PTPRE	protein tyrosine phosphatase, receptor type, E	0,38204375	2,4563472
314	AF116897	ATRN	attractin	0,34361038	2,4407513
315	AK018041	2810428J06RIK	RIKEN cDNA 2810428J06 gene	0,47020838	2,4407513
316	AK038160	MNAT1	menage a trois 1	0,38099533	2,4330275
317	NM_009036	RBPJL	recombination signal binding protein for immunoglobulin kappa J region-like(Rbpjl)	0,3145354	2,4177256
318	NM_181317	KCNS2	K+ voltage-gated channel, subfamily S, 2	0,43936175	2,4177256
319	NM_009382	THY1	thymus cell antigen 1, theta	0,22865134	2,6428761
320	NM_007788	CSNK2A1	casein kinase II, alpha 1 polypeptide	0,29777083	2,6428761
321	AK134157	5033406O09RIK	RIKEN cDNA 5033406O09 gene	0,29632264	2,562789
322	NM_025393	S100A14	S100 calcium binding protein A14	0,3033049	2,562789

323	NM_009962	PTGDR2	prostaglandin D2 receptor 2(Ptgdr2)	0,2221433	2,562789
324	AK038950	ZHX3	zinc fingers and homeoboxes 3	0,38851312	2,562789
325	AK086340	EPB4.1L3	erythrocyte protein band 4.1-like 3	0,397694	2,562789
326	NM_145959	FAM91A1	family with sequence similarity 91, member A1(Fam91a1)	0,396644	2,562789
327	NM_011804	CREG1	cellular repressor of E1A-stimulated genes 1	0,23535064	2,562789
328	NM_172564	TNS4	tensin 4	0,32933596	2,562789
329	AK052186	D330005C11RIK	RIKEN cDNA D330005C11 gene	0,3551699	2,562789
330	AK138405	D3ERTD751E	DNA segment, Chr 3, ERATO Doi 751, expressed	0,41178137	2,562789
331	XM_994725	HTRA3	HtrA serine peptidase 3	0,4029302	2,663139
332	NM_027423	POLR3B	polymerase (RNA) III (DNA directed) polypeptide B	0,33478236	2,663139
333	NM_013915	ZFP238	zinc finger protein 238	0,26592276	2,754042
334	NM_031870	MSH4	mutS homolog 4 (E. coli)	0,31282896	2,754042
335	NM_031494	ZFP275	zinc finger protein 275	0,40362698	2,754042
336	NM_024437	NUDT7	nudix (nucleoside diphosphate linked moiety X)-type motif 7	0,3688317	2,9224787
337	NM_011066	PER2	period homolog 2 (Drosophila)	0,27159828	2,9224787
338	NM_026005	2610301B20RIK	RIKEN cDNA 2610301B20 gene	0,28998226	2,9224787
339	NM_018756	TCSTV1	2-cell-stage, variable group, member 1	0,3405772	2,9224787
340	NM_010140	EPHA3	Eph receptor A3	0,341277	2,9224787
341	AK005461	TBC1D5	TBC1 domain family, member 5	0,34674937	2,9224787
342	NM_025832	NAA16	N(alpha)-acetyltransferase 16, NatA auxiliary subunit(Naa16)	0,35224184	2,9224787
343	AK032822	ITGA9	integrin alpha 9(Itga9)	0,31744772	2,9911513
344	NM_177874	F830003B07	hypothetical protein F830003B07	0,25935337	2,9911513
345	NM_177391	FAM109B	family with sequence	0,316802	2,9911513

			similarity 109, member B(Fam109b)		
346	AK031401	PRPF38A	PRP38 pre-mRNA processing factor 38 (yeast) domain containing A	0,44449255	2,9911513
347	NM_024440	DERL3	Der1-like domain family, member 3	0,27728784	2,9911513
348	NM_139309	FCMD	Fukuyama type congenital muscular dystrophy homolog (human)	0,281138	2,982556
349	AK046596	CADPS2	Ca2+-dependent activator protein for secretion 2	0,5321584	3,0578732
350	NM_080708	BMP2K	BMP2 inducible kinase	0,3376582	3,0578732
351	XM_888349	LOC433882	hypothetical gene supported by AK035725;AK051021;AK087794;BC064678	0,31138396	3,0578732
352	NM_025565	SPBC25	spindle pole body component 25 homolog (S. cerevisiae)	0,33455467	3,0578732
353	AK040124	ITFG1	integrin alpha FG-GAP repeat containing 1	0,29835412	3,0967033
354	NM_175484	CORO2B	coronin, actin binding protein, 2B	0,23680058	3,0967033
355	NM_027226	FYTTD1	forty-two-three domain containing 1	0,48463455	3,0967033
356	AK005982	H2BFM	H2B histone family, member M(H2bfm)	0,2549942	3,0967033
357	BC093505	FAM57B	family with sequence similarity 57, member B(Fam57b)	0,4834116	3,0967033
358	NM_146002	RHBDD2	rhomboid domain containing 2	0,4394378	3,0967033
359	NM_138686	CYS1	cystin 1	0,38525185	3,0967033
360	AK015725	MRO	Maestro	0,21340932	3,0967033
361	BC026481	IQSEC1	IQ motif and Sec7 domain 1	0,4648691	3,1085043
362	AK077427	MAN2C1OS	mannosidase, alpha, class 2C, member 1, opposite strand(Man2c1os)	0,2328173	3,1085043
363	NM_009356	PRSS40	protease, serine 40(Prss40)	0,3237271	3,1085043

364	NM_031382	TEX16	testis expressed gene 16	0,33411476	3,1085043
365	NM_181542	SLFN10	schlafen 10	0,34282666	3,1085043
366	AK013907	3021401L19RIK	RIKEN cDNA 3021401L19 gene	0,46639037	3,1085043
367	NM_177036	CEACAM19	CEA-related cell adhesion molecule 19	0,47385624	3,1085043
368	NM_008791	PCP4	Purkinje cell protein 4	0,42709088	3,1085043
369	NM_198127	ABI2	abl-interactor 2	0,4660605	3,1085043
370	AK018281	6430514K02RIK	RIKEN cDNA 6430514K02 gene	0,25926235	3,1085043
371	AK139501	GM428	gene model 428, (NCBI)	0,44188294	3,1085043
372	NM_023232	DIABLO	diablo homolog (Drosophila)	0,34806663	3,0753467
373	BC040756	BC040756	cDNA sequence BC040756	0,4798622	3,0753467
374	NM_019756	TUBD1	tubulin, delta 1	0,24649462	3,0753467
375	NM_020284	CTSR	cathepsin R	0,453512	3,0753467
376	NM_146176	CNOT3	CCR4-NOT transcription complex, subunit 3	0,18032686	3,2543352
377	NM_178411	ZFP334	zinc finger protein 334	0,23099491	3,2543352
378	NM_172456	EXOG	endo/exonuclease (5'-3'), endonuclease G-like(Exog)	0,5503622	3,2543352
379	NM_026201	CCAR1	cell division cycle and apoptosis regulator 1	0,49018383	3,2287107
380	NM_020583	ISG20	interferon-stimulated protein	0,41191295	3,2287107
381	NM_020507	TOB2	transducer of ERBB2, 2	0,38303724	3,2287107
382	XM_001004384	SYDE2	synapse defective 1, Rho GTPase, homolog 2 (C. elegans)	0,42241845	3,2034862
383	NM_175318	SPTY2D1	SPT2, Suppressor of Ty, domain containing 1 (S. cerevisiae)	0,40524468	3,2034862
384	NM_146809	OLFR1426	olfactory receptor 1426	0,12453806	3,2034862
385	NM_008929	DNAJC3	DnaJ (Hsp40) homolog, subfamily C, member 3	0,2560395	3,2611325
386	NM_026792	AGPAT5	1-acylglycerol-3-phosphate O-acyltransferase 5 (lysophosphatidic acid acyltransferase, epsilon)	0,15290211	3,2611325

387	NM_008424	KCNE1	potassium voltage-gated channel, Isk-related subfamily, member 1	0,24727407	3,2611325
388	NM_016909	TSNAX	translin-associated factor X	0,37635276	3,2611325
389	NM_010402	HAND2	heart and neural crest derivatives expressed transcript 2	0,3091188	3,2611325
390	NM_011890	SGCB	sarcoglycan, beta (dystrophin-associated glycoprotein)	0,58552045	3,2361748
391	NM_019944	MNX1	motor neuron and pancreas homeobox 1(Mnx1)	0,4741837	3,2361748
392	NM_026887	AP1S2	adaptor-related protein complex 1, sigma 2 subunit	0,4550875	3,2361748
393	NM_026402	ATG3	autophagy-related 3 (yeast)	0,25977564	3,2279403
394	NM_029107	FAM228A	family with sequence similarity 228, member A(Fam228a)	0,30848506	3,195417
395	BC023116	CGREF1	cell growth regulator with EF hand domain 1	0,37060818	3,195417
396	NM_007712	CLK2	CDC-like kinase 2	0,2103226	3,195417
397	NM_175194	SLC25A16	solute carrier family 25 (mitochondrial carrier, Graves disease autoantigen), member 16	0,38878947	3,195417
398	NM_181400	WDR47	WD repeat domain 47	0,44459903	3,1873884
399	AK038916	CEP120	centrosomal protein 120(Cep120)	0,3000237	3,155673
400	AK049588	NIPBL	Nipped-B homolog (Drosophila)	0,3779478	3,155673
401	BC072644	CDC14A	CDC14 cell division cycle 14 homolog A (S. cerevisiae)	0,44208017	3,155673
402	NM_011264	REV3L	REV3-like, catalytic subunit of DNA polymerase zeta RAD54 like (S. cerevisiae)	0,378029	3,155673
403	NM_011884	RNGTT	RNA guanylyltransferase and 5'-phosphatase	0,23920144	3,2272158

404	NM_022324	SDF2L1	stromal cell-derived factor 2-like 1	0,42320907	3,2272158
405	NM_008997	RAB11B	RAB11B, member RAS oncogene family	0,3808882	3,2272158
406	NM_009785	CACNA2D3	calcium channel, voltage-dependent, alpha2/delta subunit 3	0,49108523	3,211357
407	NM_199059	TBPL2	TATA box binding protein like 2(Tbpl2)	0,31718472	3,211357
408	NM_144922	HNRPUL1	heterogeneous nuclear ribonucleoprotein U-like 1	0,5429615	3,1956537
409	XM_897631	4631416L12RIK	RIKEN cDNA 4631416L12 gene	0,41057757	3,1956537
410	NM_009995	CYP21A1	cytochrome P450, family 21, subfamily a, polypeptide 1	0,28330564	3,1878595
411	AK133157	6430548M08RIK	RIKEN cDNA 6430548M08 gene	0,46655852	3,180103
412	NM_025740	CFAP36	cilia and flagella associated protein 36(Cfap36)	0,40220067	3,1193852
413	AK033703	HMGA2-PS1	high mobility group AT-hook 2, pseudogene 1	0,5604396	3,1193852
414	NM_025413	1110058A15RIK	RIKEN cDNA 1110058A15 gene	0,4491017	3,1193852
415	NM_001008502	BBS12	Bardet-Biedl syndrome 12 (human)(Bbs12)	0,24263546	3,1193852
416	NM_015797	FBXO6B	F-box only protein 6b	0,44779137	3,1193852
417	AK042393	RFC3	replication factor C (activator 1) 3	0,41551727	3,1193852
418	AK038597	2900092D14RIK	RIKEN cDNA 2900092D14 gene	0,2657141	3,1193852
419	AK008094	IGL-V1	immunoglobulin lambda chain, variable 1	0,2917273	3,1193852
420	AK158045	NAGLU	alpha-N-acetylglucosaminidase (Sanfilippo disease IIIB)	0,5841801	3,111958
421	AK049514	SLC18A1	solute carrier family 18 (vesicular monoamine), member 1	0,17033206	3,2871878
422	NM_013589	LTBP2	latent transforming growth factor beta binding protein 2	0,39903936	3,2183862

423	XM_891407	DOCK10	dedicator of cytokinesis 10	0,41727945	3,2183862
424	AK047881	SORCS2	sortilin-related VPS10 domain containing receptor 2	0,5069144	3,2183862
425	NM_028027	ARHGEF25	Rho guanine nucleotide exchange factor (GEF) 25(Arhgef25)	0,48075148	3,2183862
426	NM_009738	BCHE	butyrylcholinesterase	0,42912525	3,2183862
427	NM_009931	COL4A1	procollagen, type IV, alpha 1	0,24722211	3,2183862
428	BC059864	FAXC	failed axon connections homolog(Faxc)	0,3369929	3,2183862
429	NM_178027	VPS26B	vacuolar protein sorting 26 homolog B (yeast)	0,36919233	3,2183862
430	NM_009767	CHIC1	cysteine-rich hydrophobic domain 1	0,35398164	3,2183862
431	NM_008394	IRF9	interferon regulatory factor 9(Irf9)	0,26210743	3,3814578
432	BC029706	MIEF1	mitochondrial elongation factor 1(Mief1)	0,3969799	3,3814578
433	XM_890447	MLLT4	myeloid/lymphoid or mixed lineage-leukemia translocation to 4 homolog (Drosophila)	0,4081722	3,3504353
434	NM_011410	SLFN4	schlafen 4	0,47931868	3,3504353
435	NM_010732	LRRN2	leucine rich repeat protein 2, neuronal	0,4878737	3,3504353
436	NM_147112	OLFR559	olfactory receptor 559	0,23864828	3,3504353
437	NM_053137	PCDHB12	protocadherin beta 12	0,28670838	3,2826736
438	NM_010071	DOK2	docking protein 2	0,54055434	3,2826736
439	NM_028719	CPNE4	copine IV	0,27685776	3,2826736
440	XM_916502	SEC24B	SEC24 related gene family, member B (S. cerevisiae)	0,3786658	3,2826736
441	AK005867	IZUMO3	IZUMO family member 3(Izumo3)	0,28933313	3,2826736
442	AK036681	5830457H20RIK	RIKEN cDNA 5830457H20 gene	0,41618258	3,2826736
443	NM_001033225	PNRC1	proline-rich nuclear receptor coactivator 1	0,36049676	3,2826736
444	NM_176831	PPCDC	phosphopantothenoylcysteine decarboxylase	0,55486983	3,2826736

445	NM_144911	RPAP2	RNA polymerase II associated protein 2(Rpap2)	0,4452447	3,2826736
446	NM_010706	LGALS4	lectin, galactose binding, soluble 4	0,31004182	3,2461994
447	AK005393	1600002D24RIK	RIKEN cDNA 1600002D24 gene	0,32956177	3,2461994
448	NM_177667	TTC22	tetratricopeptide repeat domain 22	0,47258237	3,2461994
449	NM_172865	MANEA	mannosidase, endo-alpha	0,47526526	3,2461994
450	AK083496	CCNI	cyclin I	0,53631455	3,2461994
451	NM_173369	CYLD	cylindromatosis (turban tumor syndrome)	0,35098842	3,2950144
452	NM_009847	CD2AP	CD2-associated protein	0,2503716	3,2950144
453	NM_144902	SLC35A3	solute carrier family 35 (UDP-N-acetylglucosamine (UDP-GlcNAc) transporter), member 3	0,30339628	3,2950144
454	AK140938	1810026B05RIK	RIKEN cDNA 1810026B05 gene	0,37306952	3,2950144
455	NM_178608	REEP1	receptor accessory protein 1	0,42082733	3,2950144
456	NM_011403	SLC4A1	solute carrier family 4 (anion exchanger), member 1	0,46015736	3,3573656
457	NM_016694	PARK2	parkin	0,47301197	3,3573656
458	NM_015830	CAPN15	calpain 15(Capn15)	0,27908102	3,3573656
459	NM_010051	DKK1	dickkopf homolog 1 (Xenopus laevis)	0,22776048	3,350051
460	NM_008482	LAMB1	laminin B1(Lamb1)	0,46730712	3,4115047
461	NM_028934	4930452B06RIK	RIKEN cDNA 4930452B06 gene	0,37823796	3,4115047
462	NM_008136	GNL1	guanine nucleotide binding protein-like 1(Gnl1)	0,433793	3,4115047
463	NM_178395	ZDHHC2	zinc finger, DHHC domain containing 2	0,268406	3,5548363
464	XM_129366	CSPP1	centrosome and spindle pole associated protein 1	0,30940545	3,5548363
465	NM_013737	PLA2G7	phospholipase A2, group VII (platelet-activating factor acetylhydrolase, plasma)	0,43335578	3,5548363

466	AK005700	NOSIP	nitric oxide synthase interacting protein	0,34314492	3,547208
467	NM_025947	DYNLRB1	dynein light chain roadblock-type 1	0,3445403	3,7544043
468	NM_173408	DCUN1D3	DCN1, defective in cullin neddylation 1, domain containing 3 (S. cerevisiae)	0,31982046	3,7544043
469	NM_201368	XKR8	X Kell blood group precursor related family member 8 homolog	0,5723924	3,7544043
470	NM_016759	RAP2IP	Rap2 interacting protein	0,37197506	3,7544043
471	NM_173747	GPKOW	G patch domain and KOW motifs	0,31075627	3,7544043
472	AK220506	FRY	furry homolog (Drosophila)	0,5514629	3,6535628
473	NM_145216	RASL10A	RAS-like, family 10, member A(Rasl10a)	0,49235618	3,6535628
474	NM_007467	APLP1	amyloid beta (A4) precursor-like protein 1	0,25487742	3,6535628
475	NM_009364	TFPI2	tissue factor pathway inhibitor 2	0,48790053	3,6535628
476	NM_138750	PROM2	prominin 2	0,1270139	3,6535628
477	NM_033587	PCDHGA4	protocadherin gamma subfamily A, 4	0,2579723	3,6535628
478	XM_973921	LOC664994	similar to isochorismatase domain containing 2	0,3942172	3,6535628
479	BC100496	ARRDC5	arrestin domain containing 5	0,5168486	3,6535628
480	NM_145850	VMN1R188	vomeronasal 1 receptor 188(Vmn1r188)	0,34562474	3,6535628
481	NM_144941	MAP7D1	MAP7 domain containing 1(Map7d1)	0,50264615	3,6535628
482	AK081946	MTHFS	5, 10-methenyltetrahydrofolate synthetase	0,37676257	3,6535628
483	AK163649	YME1L1	YME1-like 1 (S. cerevisiae)	0,39401233	3,6535628
484	NM_028492	CCDC103	coiled-coil domain containing 103	0,38908395	3,6535628
485	NM_144873	UHRF2	ubiquitin-like, containing PHD and RING finger domains 2	0,35457212	3,6460297
486	NM_001039483	TMCO1	transmembrane and coiled-coil domains 1	0,5097584	3,6385276

487	NM_146643	OLFR1155	olfactory receptor 1155	0,52135134	3,6310563
488	NM_009630	ADORA2A	adenosine A2a receptor	0,2564151	3,5651703
489	NM_011795	C1QL1	complement component 1, q subcomponent-like 1	0,38215292	3,5651703
490	NM_172534	ANKFN1	ankyrin-repeat and fibronectin type III domain containing 1(Ankfn1)	0,52736896	3,5651703
491	AK050671	SH2D4B	SH2 domain containing 4B	0,56555426	3,5651703
492	NM_009790	CALM1	calmodulin 1	0,16211696	3,5651703
493	NM_173770	FAM69C	family with sequence similarity 69, member C(Fam69c)	0,208187	3,5651703
494	NM_173029	ADCY10	adenylate cyclase 10(Adcy10)	0,40038678	3,5651703
495	AY550908	UBR5	ubiquitin protein ligase E3 component n-recognin 5(Ubr5)	0,5243787	3,5651703
496	NM_008183	GSTM2	glutathione S-transferase, mu 2	0,3931808	3,5651703
497	NM_013707	KRTAP14	keratin associated protein 14	0,4014041	3,589899
498	NM_028055	BTBD17	BTB (POZ) domain containing 17(Btbd17)	0,35792336	3,589899
499	NM_010751	MXD1	MAX dimerization protein 1	0,45505843	3,589899
500	NM_011316	SAA4	serum amyloid A 4	0,52599895	3,589899
501	NM_133764	ATP6V0E2	ATPase, H+ transporting, lysosomal V0 subunit E2	0,2020449	3,589899
502	AK035703	SNHG20	small nucleolar RNA host gene 20(Snhg20)	0,3576988	3,589899
503	NM_133195	CELF4	CUGBP, Elav-like family member 4(Celf4)	0,35072616	3,589899
504	NM_009988	CXADR	coxsackievirus and adenovirus receptor	0,4497759	3,589899
505	NM_178795	PPIP5K1	diphosphoinositol pentakisphosphate kinase 1(Ppip5k1)	0,32483417	3,589899
506	NM_146103	TMEM185B	transmembrane protein 185B(Tmem185b)	0,36003187	3,589899

507	NM_011026	P2RX4	purinergic receptor P2X, ligand-gated ion channel 4	0,4519491	3,589899
508	XM_891200	LOC626596	similar to regulator of G-protein signalling 22	0,4444946	3,589899
509	NM_028475	UBAP2L	ubiquitin associated protein 2-like	0,57088494	3,589899
510	NM_026735	MOBKL1A	MOB1, Mps One Binder kinase activator-like 1A (yeast)	0,30222458	3,589899
511	NM_007478	ARF3	ADP-ribosylation factor 3	0,28202757	3,589899
512	AK016951	MFSD13B	major facilitator superfamily domain containing 13B(Mfsd13b)	0,23216487	3,589899
513	NM_008308	HTR1A	5-hydroxytryptamine (serotonin) receptor 1A	0,28103495	3,589899
514	NM_022418	TMUB1	transmembrane and ubiquitin-like domain containing 1	0,32766303	3,589899
515	AK013510	DMD	dystrophin, muscular dystrophy	0,51165867	3,6363897
516	AK040384	EP300	E1A binding protein p300	0,5259314	3,6363897
517	NM_011639	TRIP6	thyroid hormone receptor interactor 6	0,43021688	3,6363897
518	NM_029100	SEPN1	selenoprotein N, 1	0,29559162	3,6363897
519	XM_001001771	9630037P07RIK	RIKEN cDNA 9630037P07 gene	0,3503352	3,770257
520	AK009730	KRTAP9-3	keratin associated protein 9-3(Krtap9-3)	0,39788207	3,770257
521	NM_024406	FABP4	fatty acid binding protein 4, adipocyte	0,42190936	3,7630203
522	NM_008541	SMAD5	MAD homolog 5 (Drosophila)	0,44056177	3,7201777
523	AK020194	8030423J24RIK	RIKEN cDNA 8030423J24 gene	0,51240516	3,7201777
524	NM_026524	MID1IP1	Mid1 interacting protein 1 (gastrulation specific G12-like (zebrafish))	0,37930363	3,7201777
525	NM_212446	PSMF1	proteasome (prosome, macropain) inhibitor subunit 1	0,46362168	3,7201777

526	NM_177656	6820408C15RIK	RIKEN cDNA 6820408C15 gene	0,44546175	3,7201777
527	NM_183257	HAMP2	hepcidin antimicrobial peptide 2	0,3406584	3,7201777
528	XM_001004301	LOC668030	similar to gonadotropin inducible ovarian transcription factor 1	0,43026015	3,7645488
529	NM_009427	TOB1	transducer of ErbB-2.1	0,50155324	3,7645488
530	AK156251	ITGAL	integrin alpha L	0,31746012	3,7645488
531	NM_022981	ZFP110	zinc finger protein 110	0,39890003	3,7645488
532	NM_177319	ZFYVE27	zinc finger, FYVE domain containing 27	0,31514913	3,779995
533	AK076610	4921534A09RIK	RIKEN cDNA 4921534A09 gene	0,3683092	3,779995
534	AK082461	HGF	hepatocyte growth factor	0,5015321	3,779995
535	AK052253	2210016F16RIK	RIKEN cDNA 2210016F16 gene	0,26563537	3,779995
536	NM_147006	OLFR392	olfactory receptor 392	0,52253526	3,779995
537	NM_009796	CAPN7	calpain 7	0,48541304	3,779995
538	NM_016805	HNRPU	heterogeneous nuclear ribonucleoprotei n U	0,22208594	3,779995
539	NM_146353	OLFR706	olfactory receptor 706	0,49495953	3,779995
540	NM_013485	C9	complement component 9	0,4341826	3,8159175
541	NM_019752	HTRA2	HtrA serine peptidase 2	0,37709674	3,8159175
542	NM_134437	IL17RD	interleukin 17 receptor D	0,53799886	3,8159175
543	AK044978	EDIL3	EGF-like repeats and discoidin I-like domains 3	0,31853905	3,8159175
544	NM_146548	OLFR800	olfactory receptor 800	0,3501719	3,8159175
545	NM_172896	NALP4A	NACHT, leucine rich repeat and PYD containing 4A	0,4494239	3,8718395
546	NM_028122	SLC14A1	solute carrier family 14 (urea transporter), member 1	0,417615	3,8718395
547	NM_145940	WIPI1	WD repeat domain, phosphoinositide interacting 1	0,34998742	3,8718395
548	NM_007908	EEF2K	eukaryotic elongation factor-2 kinase	0,3877947	3,8718395
549	NM_021406	TREM1	triggering receptor	0,4543622	3,8718395

			expressed on myeloid cells 1		
550	AK136546	CDC14A	CDC14 cell division cycle 14 homolog A (S. cerevisiae)	0,49731058	3,8718395
551	NM_178901	AI467606	expressed sequence AI467606	0,31406108	3,8718395
552	NM_172466	ADAMTS18	a disintegrin-like and metallopeptidase (reprolysin type) with thrombospondin type 1 motif, 18	0,42532724	3,8718395
553	NM_027354	POC1A	POC1 centriolar protein A(Poc1a)	0,4977381	3,8718395
554	NM_172806	BTBD7	BTB (POZ) domain containing 7	0,49581012	3,8718395
555	NM_016903	ESD	esterase D/formylglutathione hydrolase	0,22289115	3,8718395
556	NM_199021	DPP10	dipeptidylpeptidase 10	0,28699756	3,8718395
557	AK084389	D230035N22RIK	RIKEN cDNA D230035N22 gene	0,47917858	3,9128296
558	AK005548	1600027J07RIK	RIKEN cDNA 1600027J07 gene	0,47709072	3,9128296
559	NM_029816	2610028H24RIK	RIKEN cDNA 2610028H24 gene	0,39801314	3,9128296
560	NM_183178	FSD1	fibronectin type 3 and SPRY domain-containing protein	0,46642548	3,9128296
561	NM_022318	POPDC2	popeye domain containing 2	0,48881766	3,993078
562	NM_025976	BFAR	bifunctional apoptosis regulator	0,54710877	3,993078
563	NM_016665	STRA13	stimulated by retinoic acid 13	0,5758857	3,993078
564	AK005822	OPLAH	5-oxoprolinase (ATP-hydrolysing)	0,3158648	3,993078
565	BY715687	4930550G17RIK	RIKEN cDNA 4930550G17 gene	0,5335803	3,993078
566	AK039208	A230107O07RIK	RIKEN cDNA A230107O07 gene	0,5241538	3,993078
567	NM_025420	LCE5A	late cornified envelope 5A	0,43828505	3,993078
568	XM_132817	ANKRD26	ankyrin repeat domain 26	0,44309217	3,993078
569	NM_178576	CPSF4	cleavage and polyadenylation specific factor 4	0,4648223	3,9720986
570	NM_009662	ALOX5	arachidonate 5-lipoxygenase	0,40475807	3,9720986

571	NM_183037	TRIM46	tripartite motif protein 46	0,5969752	3,9720986
572	NM_029752	BRI3BP	Bri3 binding protein	0,37219775	4,0995665
573	NM_026646	SLC25A22	solute carrier family 25 (mitochondrial carrier, glutamate), member 22	0,5230864	4,081154
574	NM_145995	EDRF1	erythroid differentiation regulatory factor 1(Edrf1)	0,4099976	4,081154
575	NM_177723	VSIG8	V-set and immunoglobulin domain containing 8(Vsig8)	0,5614088	4,081154
576	AK030827	ICOS	inducible T-cell co-stimulator	0,4607973	4,081154
577	Z14986	AMD1	S-adenosylmethionine decarboxylase 1	0,52171993	4,081154
578	NM_007746	MAP3K8	mitogen activated protein kinase kinase kinase 8	0,3211143	4,081154
579	NM_146243	ACTR2	ARP2 actin-related protein 2 homolog (yeast)	0,4741834	4,081154
580	NM_008796	PCTP	phosphatidylcholine transfer protein	0,48193404	4,081154
581	AK042854	NTM	neurotrimin(Ntm)	0,43105215	4,081154
582	NM_198112	OSTN	osteocrin	0,35005042	4,081154
583	NM_145822	CD3EAP	CD3E antigen, epsilon polypeptide associated protein	0,4550949	4,081154
584	NM_172718	SGSM1	small G protein signaling modulator 1(Sgsm1)	0,38331592	4,081154
585	NM_026674	APH1C	anterior pharynx defective 1c homolog (C. elegans)	0,52408355	4,0909386
586	AK011828	HIP1	huntingtin interacting protein 1	0,516387	4,0909386
587	NM_033609	MED15	mediator complex subunit 15(Med15)	0,37915492	4,0909386
588	AK083336	C920008N22RIK	RIKEN cDNA C920008N22 gene	0,40580726	4,0909386
589	NM_053250	CRIP3	cysteine-rich protein 3	0,25764775	4,0909386
590	NM_194344	SH3TC1	SH3 domain and tetratricopeptide repeats 1	0,54606485	4,0909386

591	BC082602	CCPG1	cell cycle progression 1	0,6115545	4,0909386
592	AK040404	RAVER2	ribonucleoprotein, PTB-binding 2	0,5429272	4,0909386
593	AK169738	LARP1	La ribonucleoprotein domain family, member 1	0,3915209	4,256982
594	NM_010863	MYO1B	myosin IB	0,4500529	4,256982
595	NM_029537	TMEM98	transmembrane protein 98	0,30084476	4,256982
596	AK088678	HNRPC	heterogeneous nuclear ribonucleoprotein C	0,45363948	4,256982
597	AK164057	RAB23	RAB23, member RAS oncogene family	0,5056843	4,2501698
598	NM_001030307	DKC1	dyskeratosis congenita 1, dyskerin(Dkc1)	0,47875962	4,2501698
599	AK029702	4930512B01RIK	RIKEN cDNA 4930512B01 gene	0,3846961	4,2501698
600	NM_011365	ITSN2	intersectin 2	0,4409205	4,2501698
601	NM_027741	MRO	Maestro	0,33846277	4,2501698
602	NM_177045	CC2D1B	coiled-coil and C2 domain containing 1B	0,46111393	4,2501698
603	NM_009648	AKAP1	A kinase (PRKA) anchor protein 1	0,5187829	4,2501698
604	NM_172960	ADCK5	aarF domain containing kinase 5	0,464335	4,2501698
605	NM_198162	MORC2A	microrchidia 2A	0,53174585	4,2501698
606	NM_133199	SCN4A	sodium channel, voltage-gated, type IV, alpha	0,47798523	4,2501698
607	NM_026189	EEPD1	endonuclease/exonuclease/phosphatase family domain containing 1(Eepd1)	0,441926	4,264347
608	NM_172275	TRAFD1	TRAF type zinc finger domain containing 1	0,35253245	4,264347
609	NM_027352	GORASP2	golgi reassembly stacking protein 2	0,45382512	4,264347
610	NM_008920	PRG2	proteoglycan 2, bone marrow	0,5620853	4,264347
611	NM_030033	CRISP4	cysteine-rich secretory protein 4	0,3488976	4,264347
612	AK162471	6030458C11RIK	RIKEN cDNA 6030458C11 gene	0,64997625	4,264347
613	NM_011050	PDCD4	programmed cell death 4	0,385676	4,264347
614	AK039236	A330032B11RIK	RIKEN cDNA A330032B11 gene	0,49198607	4,2435794

615	NM_146707	OLFR410	olfactory receptor 410	0,37685794	4,2435794
616	NM_172562	TADA2L	transcriptional adaptor 2 (ADA2 homolog, yeast)-like	0,55395424	4,2435794
617	XM_993279	LOC640534	similar to 60S ribosomal protein L17 (L23) (Amino acid starvation-induced protein) (ASI)	0,3770408	4,162492
618	NM_028176	CDA	cytidine deaminase	0,4922166	4,162492
619	NM_177280	B230206H07RIK	RIKEN cDNA B230206H07 gene	0,50605124	4,162492
620	NM_008591	MET	met proto-oncogene	0,51648045	4,162492
621	NM_009177	ST3GAL1	ST3 beta-galactoside alpha-2,3-sialyltransferase 1	0,3823393	4,162492
622	AK029930	A230057D06RIK	RIKEN cDNA A230057D06 gene	0,3864673	4,162492
623	AK039440	POLR3A	polymerase (RNA) III (DNA directed) polypeptide A	0,46775755	4,162492
624	AK082918	TGFBRAP1	transforming growth factor, beta receptor associated protein 1	0,43726355	4,162492
625	NM_175538	NIM1K	NIM1 serine/threonine protein kinase(Nim1k)	0,4810179	4,162492
626	NM_016979	PRKX	protein kinase, X-linked	0,38494754	4,162492
627	NM_011853	OAS1B	2'-5' oligoadenylate synthetase 1B	0,39514178	4,162492
628	NM_007804	CUX2	cut-like homeobox 2(Cux2)	0,5236876	4,162492
629	AK049847	2900052N01RIK	RIKEN cDNA 2900052N01 gene	0,34132183	4,2781057
630	NM_011358	SFRS2	splicing factor, arginine/serine-rich 2 (SC-35)	0,35698843	4,318624
631	NM_007446	AMY1	amylase 1, salivary	0,520388	4,318624
632	NM_029145	RNASE10	ribonuclease, RNase A family, 10 (non-active)	0,33453125	4,318624
